# IRX3-CDK14 axis promotes glioblastoma progression by regulating LRP6-mediated canonical Wnt/β-catenin pathway

**DOI:** 10.1038/s41419-025-08387-1

**Published:** 2025-12-23

**Authors:** Yongjia Gao, Guanghui Zhang, Yahui Yu, Jie Gao, Songtao Ren, Xiaonan Wei, Rui Yang

**Affiliations:** 1 Biomedical Laboratory, School of Medicine, Liaocheng University, Liaocheng, China; 2 Medical College, Henan University of Chinese Medicine, Zhengzhou, China; 3 Department of Neurosurgery, Liaocheng People’s Hospital, Liaocheng, China; 4 Precision Medicine Laboratory for Chronic Non-communicable Diseases of Shandong Province, Institute of Precision Medicine, Jining Medical University, Jining, China

**Keywords:** CNS cancer, Oncogenes, Ubiquitylation

## Abstract

Iroquois Homeobox 3 (IRX3), a highly conserved member of the Iroquois homeobox gene family, has been implicated in obesity through its regulation of fat mass and obesity-associated (FTO) gene. Emerging evidence indicates that IRX3 plays critical roles in the development of some cancers, but the specific functions and molecular mechanisms of IRX3 in glioblastoma (GBM) remain unknown. Here, we demonstrate that IRX3 is highly expressed in GBM and significantly correlated with poor prognosis of patients. IRX3 promotes cell proliferation, colony formation, migration, and invasion in vitro and brain tumor growth in vivo. Mechanistically, IRX3 promotes the transcription of CDK14 (Cyclin Dependent Kinase 14) by binding to its promoter, which in turn stabilizes β-catenin expression through restraining its ubiquitination degradation, thereby activating the canonical Wnt/β-catenin pathway and promoting GBM growth. In addition, we identify LRP6 (LDL receptor-related protein 6) as a crucial regulatory factor in maintaining IRX3-mediated stabilization of β-catenin. Our results demonstrate that IRX3 serves as a promising biomarker for patients with GBM, and targeting the IRX3-CDK14-LRP6 axis may represent a viable treatment approach for GBM.

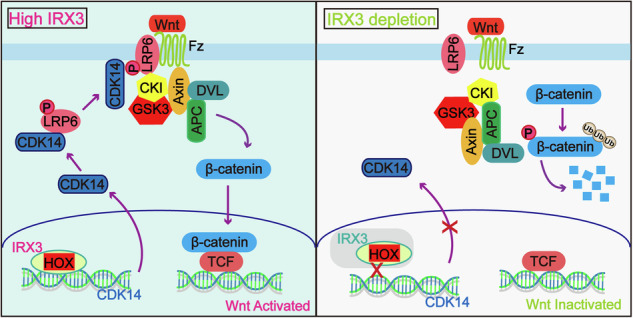

## Introduction

Glioblastoma (GBM) is the most aggressive and common primary malignancy of the central nervous system (CNS) and has a poor prognosis [1]. GBM exhibits diffuse infiltrative growth into adjacent cerebral parenchyma, which precludes complete surgical excision and results in residual neoplastic tissue postoperatively, leading to a very high recurrence rate [2]. In recent years, despite significant progress in the comprehensive treatment of GBM due to the emergence of modern therapies such as immunotherapy and virotherapy, the prognosis for GBM remains poor, with a median survival time of only about 9 months and a 5-year survival rate of 7.1% [3–6]. Currently, various molecular markers such as isocitrate dehydrogenase (IDH1), epidermal growth factor receptor (EGFR), O6-methylguanine-DNA methyltransferase (MGMT) promoter methylation and telomerase reverse transcriptase (TERT) promoter are utilized clinically to assess patient prognosis and drug sensitivity [7]. However, the key molecular mechanisms driving the invasive growth of gliomas remain unclear. Therefore, identifying new biomarkers and exploring novel therapeutic targets for GBM treatment are urgent issues that need to be addressed, which have significant clinical translational value for improving patient prognosis.

IRX3 is a highly conserved transcription factor that belongs to the Iroquois homeobox family, and the encoded protein contains a homology domain consisting of 61-amino acids [8]. IRX3 is crucial for several biological functions, such as embryonic development, neurogenesis and organ differentiation [9–11]. Previous studies have focused on the regulatory role of IRX3 in obesity, where IRX3 promotes the transcription of pro-inflammatory cytokines, thereby inhibiting adrenergic signaling in adipocytes and thus inhibiting lipolysis [12, 13]. Recent studies have revealed that IRX3 acts as a key modulator of cancer development. IRX3 is abnormally expressed in T-cell acute lymphoblastic leukemia and correlated with patient prognosis [14]. In breast cancer, IRX3 serves as an independent prognostic factor and enhances migratory and invasive capacities through transcriptional activation of pro-metastatic genes, including SNAI1 and Twist1 [15]. High expression of IRX3 is associated with treatment failure due to drug resistance in breast cancer cells, and it is hypothesized that IRX3 could be as a potential tumor marker for prognostic diagnosis and treatment of HER2^+^ breast cancer patients [16]. Previous study has showed that IRX3 is abnormally highly expressed in low-grade glioma patients and is correlated with poor outcome. Nonetheless, the specific mechanism of IRX3 in GBM progression is unclear, and further studies are needed to determine whether IRX3 is a potential drug target for GBM.

The evolutionarily conserved Wnt signaling cascade is initiated via stereotypical interactions between Wnt ligands and Frizzled (FZD) receptor family members [17]. This pathway is broadly subdivided into two distinct branches: the canonical Wnt pathway and the non-canonical Wnt pathway [18]. In GBM microenvironment, paracrine Wnt3a ligand derived from neoplastic cells activates Wnt/β-catenin signaling pathway. This activation promotes M2-like polarization of tumor-associated microglia, which enhances microglia-glioma crosstalk via CX3CL1-CX3CR1 axis activation and leads to glioma progression and poor prognosis [19]. Meanwhile, canonical Wnt/β-catenin signaling inhibits temozolomide (TMZ) delivery by modulating endothelial cell transcytosis. Blocking the Wnt/β-catenin pathway enhances tumor vascular transcytosis to promote TMZ delivery and increases GBM chemosensitivity to TMZ [20]. The activation of Wnt/β-catenin signaling typically involves the LRP5/6 co-receptor binding to FZD receptors. This interaction facilitates the dissociation of glycogen synthase kinase-3β (GSK-3β) from the APC/Axin destruction complex, thereby stabilizing β-catenin through impaired proteasomal degradation [21]. However, the precise molecular mechanisms governing this regulatory cascade in GBM remain incompletely understood.

In this research, we demonstrate that IRX3 is excessively expressed in GBM and significantly correlated with a worse prognosis of patients. Knockdown of IRX3 significantly attenuates GBM cell proliferation, colony formation, migration, and invasion, brain tumor growth, and these oncogenic phenotypes were restored upon ectopic IRX3 overexpression. Mechanically, our results reveal that IRX3-mediated oncogenic effects are induced by the activation of canonical Wnt signaling through impaired proteasomal degradation of β-catenin. This is regulated by IRX3-mediated transcriptional activation of CDK14, which then phosphorates LRP6 and stabilizes β-catenin through impaired proteasomal degradation. Overall, our findings demonstrate a novel mechanism by which IRX3-CDK14-LRP6 axis activates the canonical Wnt/β-catenin pathway and promotes GBM progression, providing a promising therapeutic strategy for GBM patients.

## Materials and methods

### Clinical patient samples

The glioma tissue microarray (N109Ct01a) was purchased from Xi’an Zhongke Guanghua Bioaitech Co., Ltd. (Xi’an, China). Fourteen GBM specimens and 4 normal brain tissues were collected in Liaocheng People’s Hospital with the participants’ written informed consent. Online patient data were downloaded from TIMER2 (http://timer.cistrome.org/), R2 (https://hgserver1.amc.nl/cgi-bin/r2/main.cgi) and GlioVis (https://gliovis.bioinfo.cnio.es/) database. The project was approved by the Research Ethics Committee of Liaocheng University.

### Immunohistochemistry (IHC) staining

The TMA, GBM tissues, and mouse brains were embedded in paraffin and sectioned into slices that are 5 μm thick, after which the paraffin sections underwent a process of dewaxing and hydration. The IRX3, CDK14 and β-catenin antibodies (1:200) were used for IHC staining, which was performed as described previously [22]. The reagents and antibodies can be found in Supplementary Table S1.

### Cells and cell culture

The human brain astrocytes SVGp12, 293FT, and glioma cell line U251 were purchased from the National Infrastructure of Cell Line Resource (Shanghai, China). The GBM cell lines LN229, A172, U87-MG, U118-MG, LN18, and U138-MG were acquired from ATCC (American Type Culture Collection). Cell lines were verified via short tandem repeat (STR) analysis and tested for mycoplasma contamination using PCR. GBM cell lines were cultured in Dulbecco’s Modified Eagle’s Medium (DMEM) with 10% FBS. Primary GBM cells were isolated from GBM specimens. Briefly, the specimens were subjected to mincing and subsequently digested using a solution containing dispase I, collagenase, and DNase I for a duration of 30 min. Following digestion, the resultant cell suspension was passed through a 70 μm cell strainer. Primary GBM cells were grown in DMEM/F12 that was enriched with 10% FBS, 2% B27 and 1% NEAA.

### Plasmids, transfection and generation of stable cell lines

The small-hairpin RNAs, specifically shCtrl, shIRX3, and shLRP6, were designed on Genetic Perturbation Platform (GPP) and subsequently synthesized by Sangon Biotech (Shanghai, China) and cloned into pLKO.1 vector. Flag-IRX3 full length, Flag-IRX3 HOX deletion and CDK14 were constructed into the lentiviral vector pCDH-CMV-MCS-EF1-Neo, which were purchased from YouBiotech (Changsha, China). Transfection and lentivirus infection were performed as described previously [23]. Stable cells were selected with G418 or puromycin for 5 days. Primers are listed in Supplementary Table S2.

### Cell proliferation

LN229 (1 × 10^3^ cells/well) and GBM02 (2 × 10^3^ cells/well) cells with IRX3 silencing or rescued IRX3 were seeded into 96-well cell culture plates. CCK-8 assay was carried out as described previously to examine the ability of cell proliferation [23].

### Edu staining

A total of 2 × 10^4^ cells/well were placed in a 24-well cell culture plate and cultured 12 h, Edu (Beyotime, Shanghai, China) staining assay was performed as manufacturer’s protocol. Briefly, cells were initially incubated with 10 mM Edu for a duration of 2 h. This was succeeded by a 15-min incubation with 4% paraformaldehyde (PFA), followed by a 10-min treatment with 0.3% Triton X-100. Subsequently, cells were treated with 5% bovine serum albumin (BSA) for 1 h. Prior to microscopic imaging for analysis, the nuclei were stained with DAPI for 30 min. The percentage of Edu was determined by analyzing a minimum of 3 microscopic fields (Olympus, Tokyo, Japan).

### Colony formation assay

Soft agar assay was employed to detect the ability of colony formation. A total of 2 × 10^3^ stable shCtrl or shIRX3 cells were mixed with 0.3% Noble agar in DMEM medium supplemented with 10% FBS and then transferred into 6-well plates that had a solidified base layer of 0.6% Noble agar in DMEM medium with 10% FBS. The colonies were photographed and documented after a period of 14–21 days.

### Quantitative real-time PCR (qRT-PCR)

Total RNA was extracted from the cells by using Trizol reagent and then converted into cDNA with the M5 Sprint qPCR RT kit, which includes a gDNA remover (Mei5 Biotech, Beijing, China). 2×M5 SYBR Premix is used for qRT-PCR (Mei5 Biotech, Beijing, China). The results were determined utilizing the ΔΔCt method at three independent experiments, employing GAPDH as the internal control.

### Gene Set Enrichment Assay (GSEA)

Data from glioma patients were sourced from the Chinese Glioma Genome Atlas (CGGA). The data underwent normalization, and significance was evaluated using ANOVA, resulting in the division into two groups: high IRX3 and low IRX3. GSEA was carried out by using GSEA 4.4.0 as previously described [22].

### Transwell assay

Cell migration and invasion tests were conducted using transwell chambers that have an 8 µm pore diameter (Beyotime, Shanghai, China). For the invasion assays, the membrane was coated with Matrigel (BD Biosciences). The lower chamber was filled with DMEM medium supplemented with 10% FBS, while the upper chamber was populated with cells suspended in serum-free DMEM medium. After incubation periods of 24 or 48 h, the stably GBM Cells were treated with 4% paraformaldehyde for 15 min for fixation, and then stained with crystal violet. The images were obtained via microscopy (Olympus, Tokyo, Japan).

### Immunoblot and immunoprecipitation analysis

For immunoblotting and immunoprecipitation assay, protein extracts were obtained by using cell lysis buffer (Beyotime, Shanghai, China). For the co-immunoprecipitation procedure, the supernatant obtained from the cell lysate was incubated with the designated antibodies for a duration of 12 h at a temperature of 4 °C, followed by a 2-h incubation with Protein A/G beads (Beyotime, Shanghai, China) at 4 °C. The proteins that were immunoprecipitated were subsequently eluted from the beads either by boiling at 95 °C for 10 min. In the immunoblotting process, proteins were separated using FuturePAGE™ (ACE biotechnology, Shanghai, China) and transferred to 0.45 mm PVDF membranes (Millipore). Following a blocking step, the membranes were incubated with the appropriate primary antibodies, followed by secondary antibodies. Clean-Blot IP (Thermo, Shanghai, China) was used to eliminate the interference of heavy and light chains for immunoprecipitation detection.

### Ubiquitination assay

For the ubiquitination assay, the recombinant plasmids shCtrl, shIRX3, CDK14, shLRP6, Flag-IRX3 and CDK14 inhibitor FMF-04-159-2 (MedChemExpress, Shanghai, China) together with HA-UB were transfected into GBM cells. Following a 48-h incubation period, MG-132 (50 μg/ml, MedChemExpress, Shanghai, China) was administered into the culture medium for an additional 8 h prior to cell harvesting. Subsequently, the cells were lysed, and the procedure was conducted in accordance with the established protocol utilized for co-immunoprecipitation.

### Turnover assay

For turnover assay, the recombinant plasmids Empty vector, Flag-IRX3, shCtrl, shIRX3 or shIRX3 together with CDK14 were transfected into GBM cells for 48 h. Then, cells were subjected to treatment with cycloheximide (CHX) at 100 µg/ml. Following this treatment, the cells were harvested, lysed, and protein extracts were analyzed via immunoblotting.

### Chromatin immunoprecipitation

The Chromatin Immunoprecipitation (ChIP) assay was conducted in accordance with previously established protocols [22]. The chromatin was subjected to immunoprecipitation using antibodies that listed in Supplementary Table S1. The sequences utilized for ChIP PCR are detailed in Supplementary Table S2.

### Luciferase reporter assay

The wild-type and mutated promoters of CDK14 were cloned into the pGL3-Basic vector. LN229 cells, which stably express either shCtrl or shIRX3, underwent transfection with the promoter reporter constructs or pGL3-Basi. After 48 h, cells were lysed using Renilla-Lumi buffer. The luciferase activities were subsequently measured in accordance with the manufacturer’s guidelines (Promega, E1910, Beijing, China).

### Animal studies

Female BALB/c nude mice were procured from the Beijing Huafukang Biotechnology and subsequently housed in a specific pathogen-free (SPF) environment. To establishing intracranial xenograft model, 1 × 10^5^ LN229 cells stably express shCtrl, shIRX3, and shIRX3/CDK14 were into nude mice (randomly divided into 9 mice/group) as described previously [23]. Three animals from each group were euthanized 14 days following the injection of cells, the brains of the mice were collected, subjected to fixation in 4% formaldehyde, and subsequently embedded in paraffin. H&E Staining and IHC were performed to assess tumor formation and phenotype. The remaining six mice within each subgroup were observed for their survival rates. All mice were euthanized under anesthesia following the observation of two or more of the established humane endpoints. The use and experiment of animals in this study received approval from the Scientific Research Ethics Committee of Liaocheng University.

### Statistical analysis

All quantitative data are presented as the mean ± standard deviation (SD) of three independent experiments. The statistical analyses were conducted by using GraphPad Prism 9.5.1. A comparative analysis involving two groups was performed by using the unpaired two-tailed Student’s *t* test. In instances where comparisons encompassed multiple groups and/or conditions, one-way or two-way analysis of variance (ANOVA) was applied. *p* < 0.05 was considered for statistical significance.

## Results

### IRX3 is up-regulated in GBM and correlated with poor prognostic outcomes

**Fig. 1:**
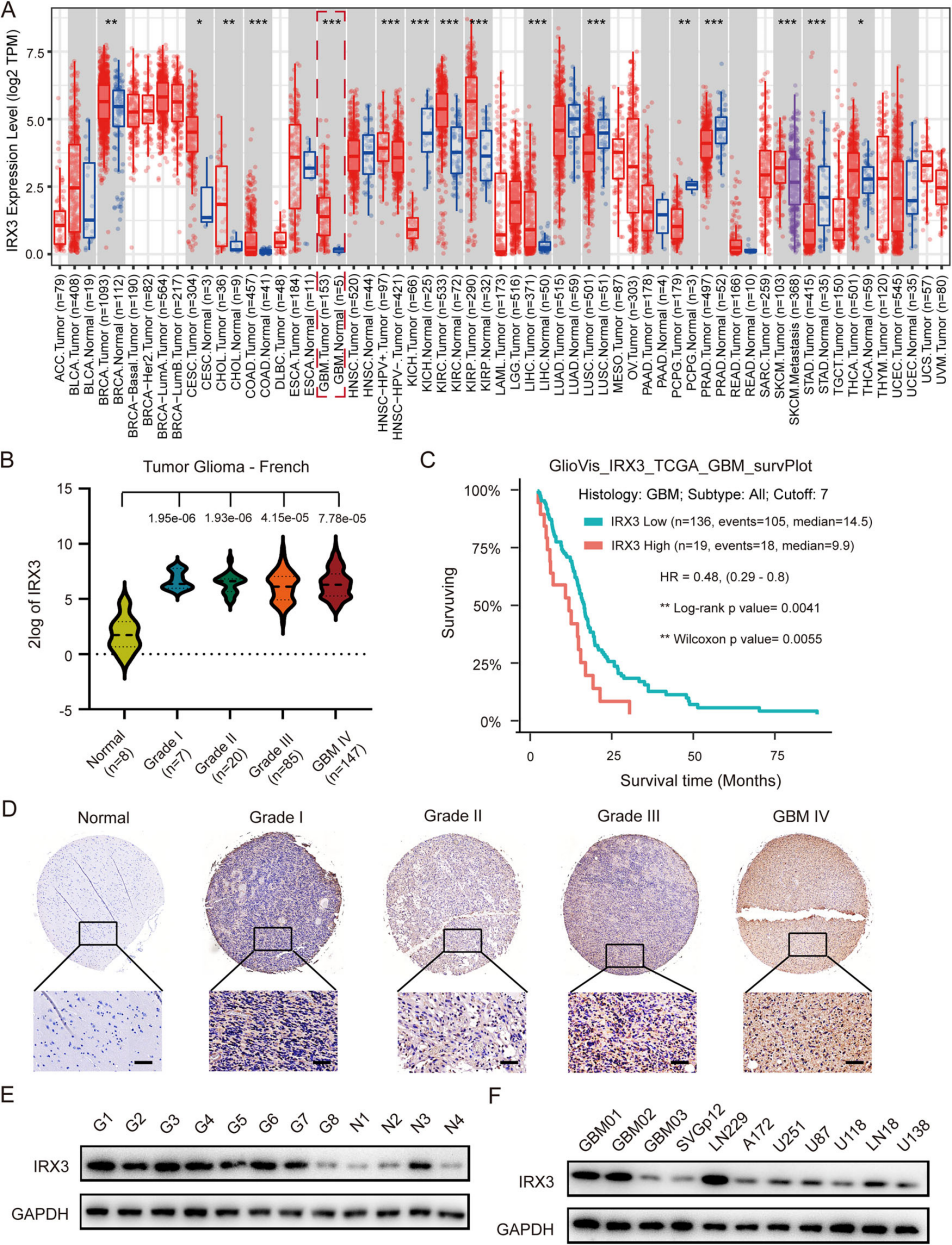
IRX3 is up-regulated in GBM and predicts poor prognostic outcomes. **A** Expression of IRX3 in GBM and many other tumor types in TIMER2 dataset. **B** Expression of IRX3 in normal tissues, low-grade glioma, and GBM in the French database. **C** Kaplan–Meier analysis of overall survival from GlioVis_TCGA GBM patients with high IRX3 expression or low IRX3. **p < 0.01. **D** Representative immunohistochemistry images of IRX3 staining in glioma TMA. **E** Immunoblotting analysis of IRX3 protein levels in GBM specimens and normal brain tissues. **F** Immunoblotting analysis of IRX3 protein levels in primary GBM cells, GBM cell lines and normal glia cells.

To determine the expression status of IRX3 in GBM, we first analyzed the patients’ data using TCGA dataset. Database analysis indicated that IRX3 was up-regulated in GBM as well as some other cancers (Fig. 1A). Subsequently, we analyzed the expression of IRX3 in different grade glioma according to R2 French database. The results demonstrated that IRX3 was up-regulated across all glioma subtypes relative to non-neoplastic brain tissue (Fig. 1B). In addition, we explored the relationship between IRX3 expression and MGMT promoter methylation status. The results showed that the expression of IRX3 was negatively correlated with IDH mutations and MGMT promoter methylation status in low-grade gliomas but not in GBM, which explains why IRX3 overexpression was correlated with a poor outcome even in low-grade gliomas (Supplementary Fig. S1A–D). The prognostic significance of IRX3 in GBM was analyzed by using the GlioVis-TCGA database. The results showed that heightened IRX3 expression levels exhibit a significant correlation with poorer clinical outcomes (Fig. 1C). To confirm the results of the online database, the expression of IRX3 was subsequently detected by performing IHC staining using glioma tissue microarray (TMA). The results further demonstrated that IRX3 expression levels were positively correlated with the progression of glioma grade (Fig. 1D). Additionally, the expression of IRX3 was also detected in local GBM patients and GBM cells by using immunoblot. Consistent with the findings of the TMA, the expression levels of IRX3 were markedly elevated in glioblastoma (GBM) specimens, cell lines, and primary GBM cells when compared to normal brain tissues and glial cells (Fig. 1E, F). All these results indicate that IRX3 serves as an adverse prognostic indicator in GBM patients.

### Knockdown of IRX3 inhibits cell proliferation in GBM

**Fig. 2:**
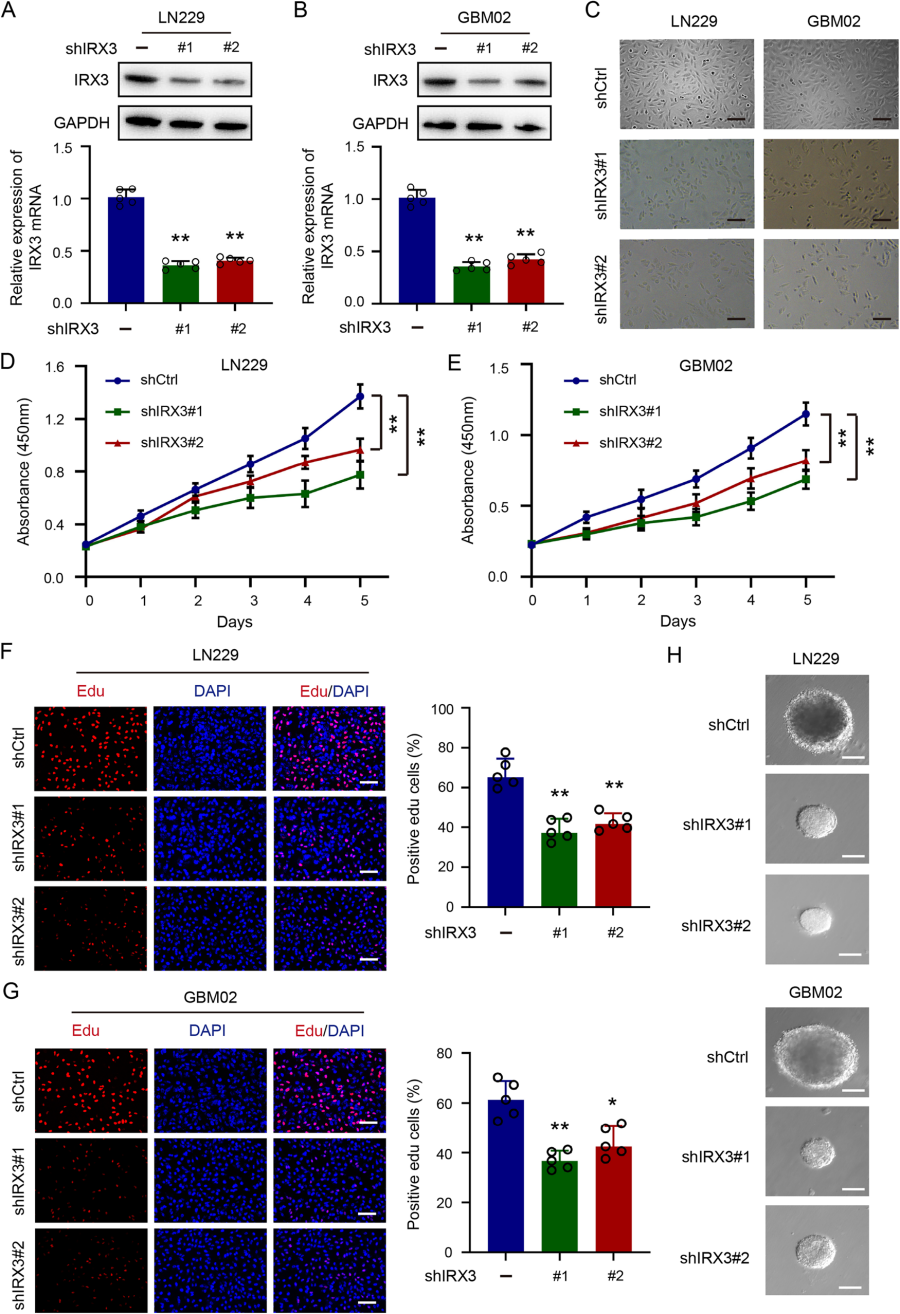
Knockdown of IRX3 inhibits cell proliferation in GBM. **A**, **B** Immunoblotting analysis and qRT-PCR analysis of IRX3 expressions in LN229 and GBM02 cells stably expressing IRX3 shRNA or a control shRNA. **C** Representative microscopical images of GBM cells with or without IRX3 knockdown. **D**, **E** Cell viabilities of GBM cells with or without IRX3 knockdown were determined by CCK-8 assay. Data were presented as means and SD (*n* = 3), **p < 0.01. **F**, **G** Representative immunofluorescence images and quantifications of Edu staining in GBM cells with IRX3 knockdown or control. **H** Representative microscopical images of colony formation in GBM cells with IRX3 knockdown or control.

To examine the biological functions of IRX3 in the progression of GBM, stable GBM cell knocking down IRX3 were constructed by using shRNAs and lentiviral infection. Immunoblotting and quantitative PCR analyses demonstrated a marked reduction in the expression levels of IRX3 in both LN229 and GBM02 cell lines (Fig. 2A, B). GBM cells with IRX3 depletion showed marked morphological alterations and a significant reduction in cell number (Fig. 2C). In addition, the proliferation ability of the cells was determined using CCK-8 assay. The results demonstrated that the silencing of IRX3 markedly inhibited the proliferation ability of GBM cells (Fig. 2D, E). The Edu incorporation assay was subsequently performed to assess DNA synthesis and the results revealed that DNA synthesis was obviously reduced in IRX3 silencing GBM cells (Fig. 2F, G). Furthermore, soft agar assay was employed to detect the capacity for colony formation in cells with IRX3 knockdown. The findings demonstrated that silencing of IRX3 significantly reduced the dimensions of clones (Fig. 2H). Taken together, our results demonstrated that IRX3 could promote cell growth in GBM.

### Knockdown of IRX3 restrains cell migration, invasion, and Wnt/β-catenin pathway in GBM

**Fig. 3:**
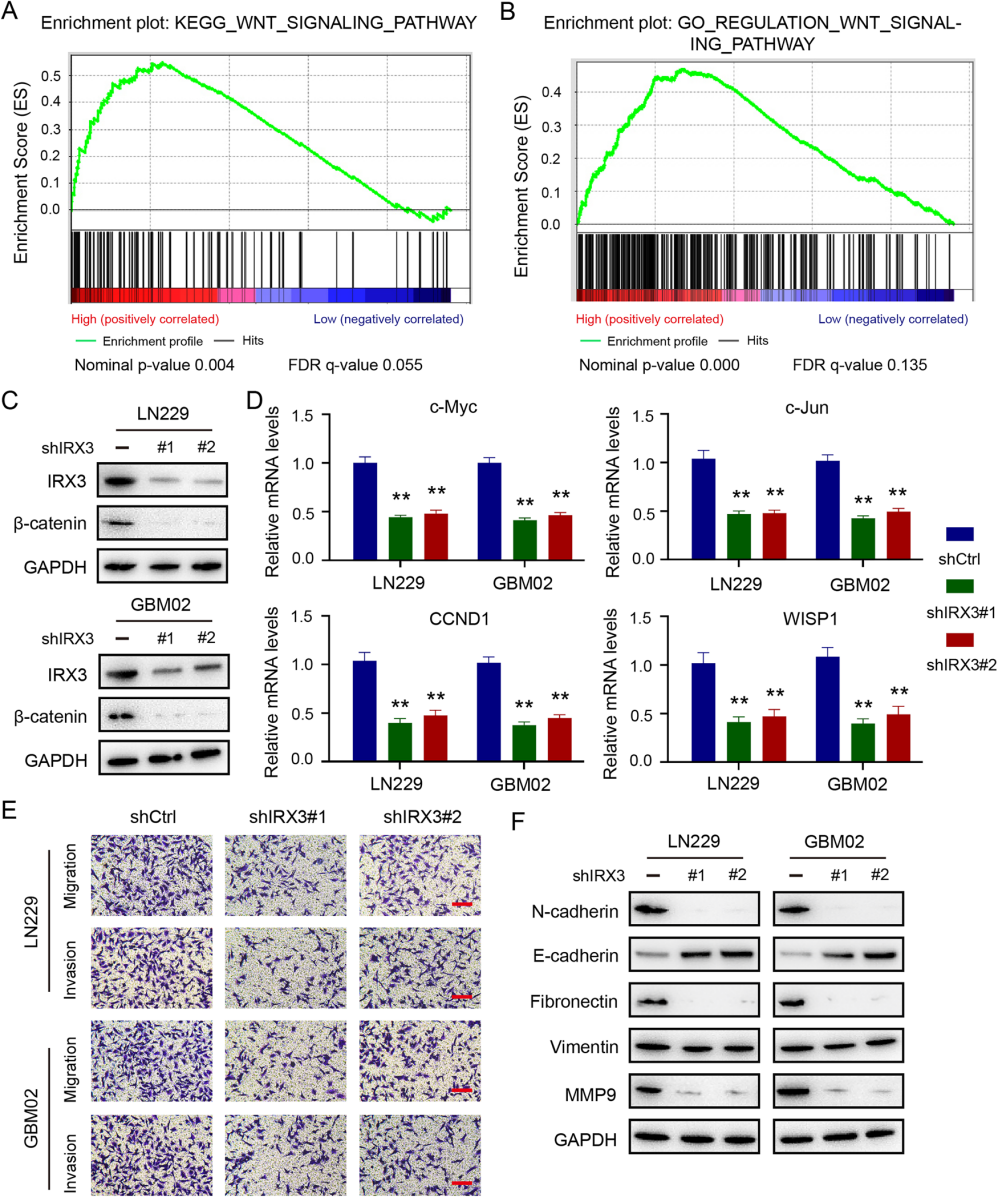
Knockdown of IRX3 suppresses cell migration, invasion and Wnt/β-catenin signaling pathway in GBM. **A**, **B** GSEA of CGGA glioma patient data showed significant enrichment of gene sets involved in the Wnt pathway with the abundant genes being upregulated (cutoff: median). **C** Immunoblotting analysis of IRX3 and β-catenin expressions in GBM cells with IRX3 knockdown or control. **D** qRT-PCR analysis of c-Myc, c-Jun, CCND1 and WISP1 mRNA levels in GBM cells with IRX3 knockdown or control. Data were analyzed with Student’s *t* test and shown as means and SD (*n* = 3), **p < 0.01. **E** Representative microscopical images of transwell assay that detected migration and invasion of GBM cells with IRX3 knockdown or control. **F** Immunoblotting analysis of the indicated proteins in GBM cells with IRX3 knockdown or control.

To further explore the biological roles of IRX3 in GBM, we obtained glioma patient data from CGGA and performed GSEA. The results indicated that elevated expression of IRX3 was associated with an increased expression of numerous genes involved in regulating Wnt signaling pathway (Fig. 3A, B). Immunoblot assay showed that knockdown of IRX3 significantly decreased the expression of β-catenin (Fig. 3C), which is the key downstream component of the canonical Wnt signaling pathway. Moreover, the mRNA levels of Wnt target genes including c-Myc, c-Jun, CCND1, and WISP1 were also reduced in IRX3 depletion cells (Fig. 3D). The above results demonstrated that IRX3 could regulate canonical Wnt/β-catenin pathway in GBM cells. The activation of Wnt/β-catenin pathway promotes the epithelial-mesenchymal transition (EMT) associated with tumorigenesis. Transwell assays were conducted to evaluate the impact of IRX3 on the metastasis of GBM cells. The findings indicated that knockdown of IRX3 significantly restrained the cell migration and invasion (Fig. 3E). Additionally, the expression of metastasis-related proteins N-cadherin, fibronectin, and MMP9 were obviously decreased in IRX3-silenced cells. In contrast, the expression of E-cadherin was increased after IRX3 knockdown (Fig. 3D). These results suggested that IRX3 might promote EMT and cell migration by regulating canonical Wnt/β-catenin pathway in GBM.

### IRX3 is essential for cell proliferation, migration, and invasion in GBM

**Fig. 4:**
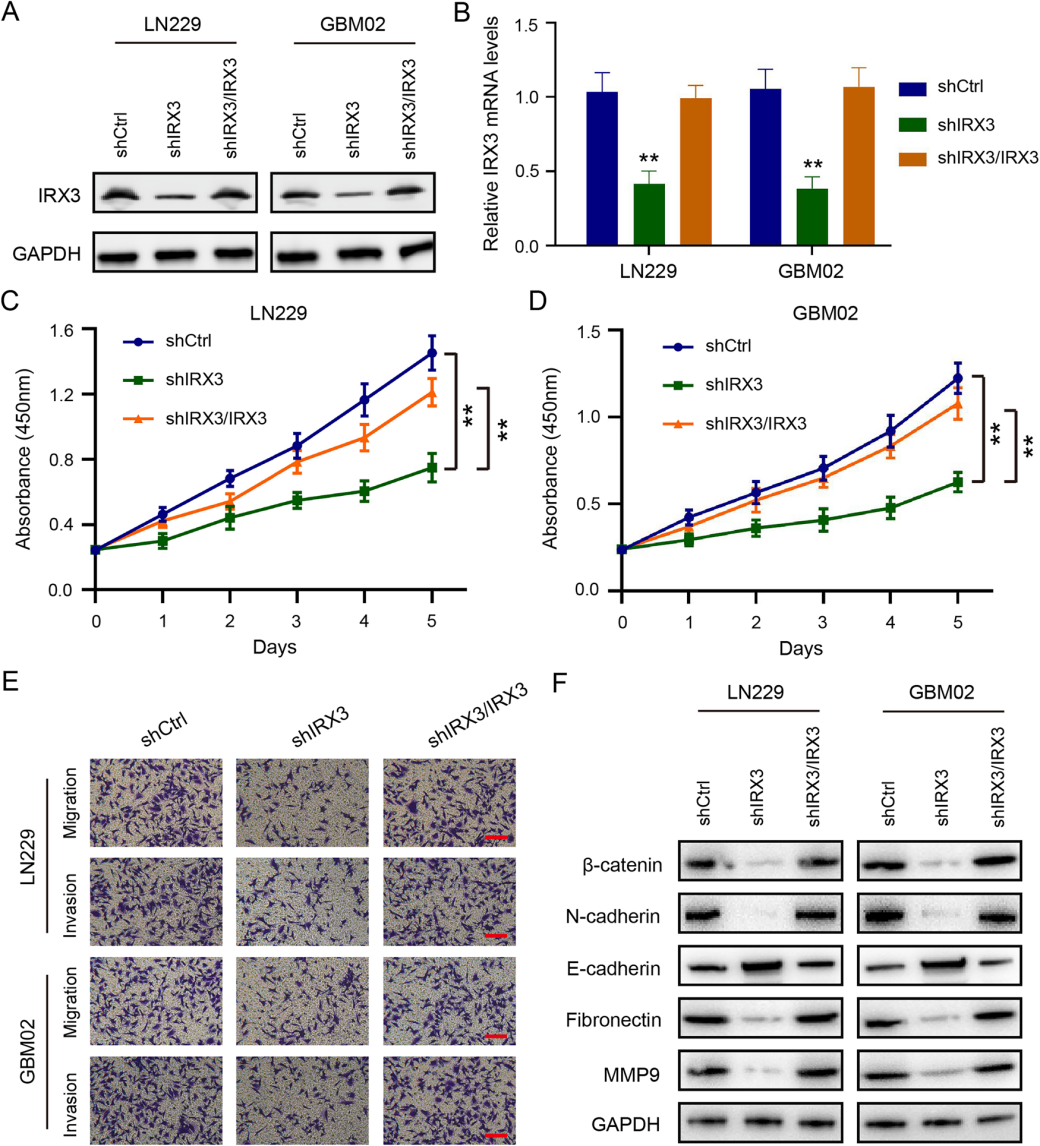
IRX3 is essential for cell proliferation, migration and invasion in GBM. **A**, **B** Immunoblotting analysis and qRT-PCR analysis of IRX3 expressions in GBM cells stably expressing shCtrl, shIRX3 with or without reconstituted expression of IRX3. Data were presented as means and SD (*n* = 3), **p < 0.01. **C**, **D** CCK-8 assay that detected cell viabilities of GBM cells stably expressing shCtrl, shIRX3 with or without reconstituted expression of IRX3. Data were presented as means and SD (*n* = 3), **p < 0.01. **E** Representative microscopical images of the transwell assay that detected migration and invasion of GBM cells stably expressing shCtrl, shIRX3 with or without reconstituted expression of IRX3. **F** Immunoblotting analysis of the indicated proteins in GBM cells stably expressing shCtrl, shIRX3 with or without reconstituted expression of IRX3.

To verify that the biological effects induced by IRX3 knockdown were not due to off-target, we restored the expression of IRX3 in IRX3-silenced cells. Immunoblot and quantitative PCR assays showed that the expression of IRX3 was restored in LN229 and GBM02 cells (Fig. 4A, B). CCK-8 assays showed that cell proliferation was obviously rescued by reconstituted expression of IRX3 (Fig. 4C, D). Transwell assays were then used to determine the abilities of cell migration and invasion in response to overexpression of IRX3 in GBM cells with IRX3 knockdown. The findings indicated that the reestablishment of IRX3 expression significantly restored the abilities of cell migration and invasion (Fig. 4E). In line with these results, immunoblot showed that expressions of N-cadherin, Fibronectin, and MMP9 were restored, while E-cadherin expression was abolished after reconstituted expression of IRX3 (Fig. 4F).

Our results indicated that IRX3 is essential for GBM cell proliferation, migration, and invasion.

### IRX3 stabilizes β-catenin expression by restraining its ubiquitination degradation

**Fig. 5:**
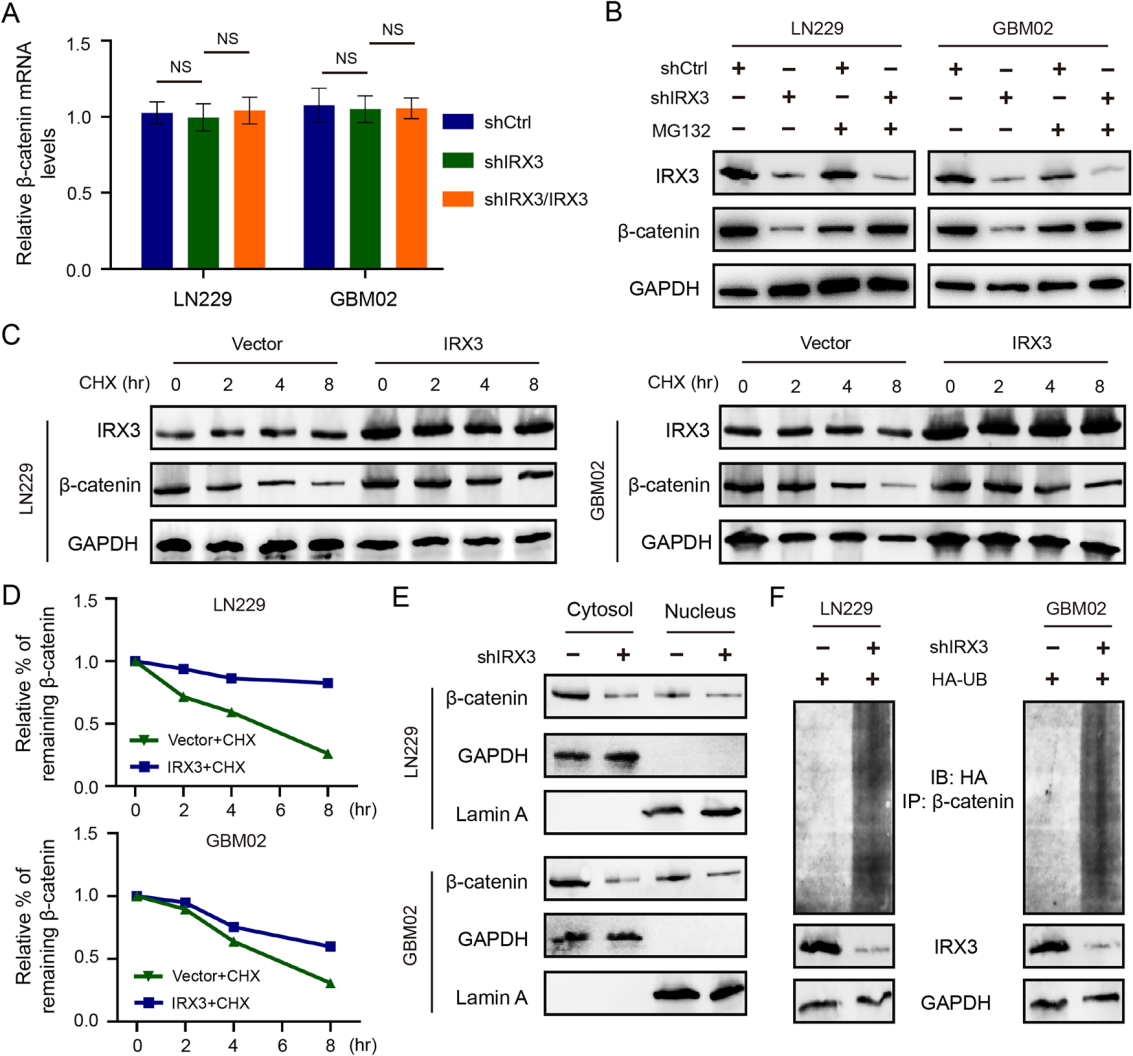
IRX3 stabilizes β-catenin expression by restraining its ubiquitination degradation. **A** qRT-PCR analysis of β-catenin in GBM cells stably expressing shCtrl, shIRX3 with or without reconstituted expression of IRX3. Data were presented as means and SD (*n* = 3), NS no significance. **B** Immunoblotting analysis of IRX3 and β-catenin expressions in IRX3-silenced cells treated with or without MG132 for 8 h. **C** Immunoblotting analysis of IRX3 and β-catenin expressions in IRX3-overexpressed cells treated with or without CHX for the indicated times. **D** The turnover rate of β-catenin in GBM cells overexpressing IRX3. **E** Immunoblotting analysis of β-catenin expression of IRX3-silenced cells in cytosol and nucleus. **F** Ubiquitination assay of β-catenin in IRX3-silenced cells treated with MG132. Immunoblotting with the indicated antibodies was performed on equal amounts of cell lysates.

Given that IRX3 functions as a transcription factor, we investigated its potential role in regulating the expression of β-catenin through the modulation of its transcriptional activity. Unexpectedly, qRT-PCR results indicated that IRX3 did not change the mRNA levels of β-catenin (Fig. 5A), suggested that IRX3 inhibited the degradation of β-catenin. The ubiquitin-proteosome system serves as the primary mechanism for the degradation of intracellular proteins. Hence, we detected whether IRX3 regulates the ubiquitination degradation of β-catenin. The results showed that treatment with MG132 obviously rescued the protein levels of β-catenin in IRX3-silenced GBM cells (Fig. 5B). Furthermore, the application of the de novo protein synthesis inhibitor cycloheximide (CHX) to assess the turnover rate of β-catenin demonstrated that the degradation rate of β-catenin was significantly reduced in GBM cells that overexpressed IRX3 (Fig. 5C, D). The ubiquitin-mediated degradation of β-catenin prevents its translocation into the nucleus, thereby inhibiting its ability to activate transcription. Our findings also revealed that the silencing of IRX3 reduced the expression of β-catenin in the nucleus (Fig. 5E). The ubiquitination assay was employed to verify that the knockdown of IRX3 resulted in an enhancement of the ubiquitination-mediated degradation of β-catenin (Fig. 5F). All these results demonstrated that IRX3 stabilizes the expression of β-catenin by restraining its ubiquitination degradation.

### IRX3 is a direct transcriptional activator of CDK14

**Fig. 6:**
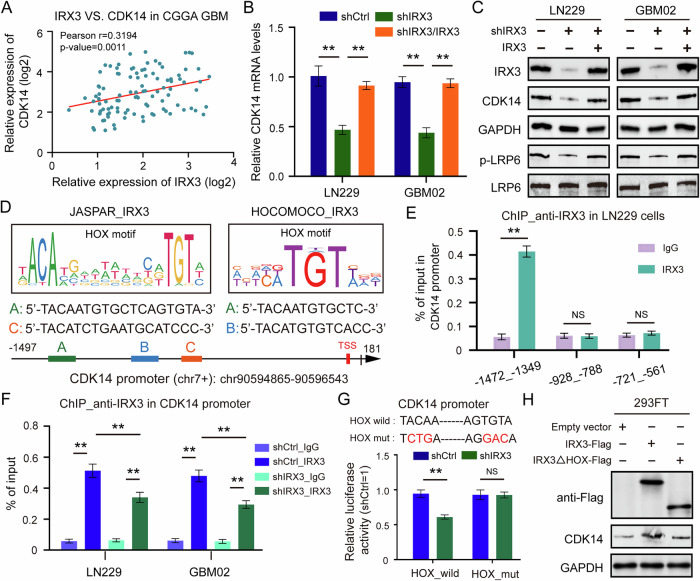
IRX3 is a direct transcriptional activator of CDK14. **A** The correlation of IRX3 expression with CDK14, GLUT1 in CGGA GBM patients’ data. **B** qRT-PCR analysis of CDK14 in GBM cells stably expressing shCtrl, shIRX3 with or without reconstituted expression of IRX3. Data were presented as means and SD (*n* = 3), **p < 0.01. **C** Immunoblotting analysis of the indicated proteins in GBM cells stably expressing shCtrl, shIRX3 with or without reconstituted expression of IRX3. **D** The predicted sequences of IRX3 HOX motif in *CDK14* promoter by JASPAR and HOCOMOCO database. **E** ChIP-qPCR analysis of IRX3 protein levels at different regions of *CDK14* promoter in LN229 cells. Data were presented as means and SD (*n* = 3), **p < 0.01. **F** ChIP-qPCR analysis of IRX3 protein levels at the promoter region of *CDK14* including −808 to −796 in GBM cells expressing shCtrl or shIRX3. Data were presented as means and SD (*n* = 3), **p < 0.01. **G** Luciferase promoter/reporter constructs containing the wild *CDK14* promoter or HOX motif mutant were co-transfected with shCtrl or shIRX3 into HEK293FT cells. The luciferase activities were detected after 48 h. Data were presented as means and SD (*n* = 3), **p < 0.01, NS no significance. **H** Immunoblotting analysis of the indicated proteins in 293FT cells expressing Flag-IRX3 full length or Flag-IRX3 HOX deletion.

In order to explore the underlying mechanisms by which IRX3 activates the Wnt/β-catenin signaling pathway, we analyzed GBM patient data from CGGA. The result identified a significant positive correlation between IRX3 and CDK14 (Fig. 6A), which mediates the phosphorylation of LRP6 to activate the Wnt/β-catenin pathway [24]. Meanwhile, knockdown of IRX3 decreased both mRNA and protein levels of CDK14 and phosphorylation level of LRP6, these effects were rescued by reconstituted expression of IRX3 (Fig. 6B, C). To further determine whether IRX3 directly bind to the promoter region of *CDK14*, we firstly identified three potential binding sites for IRX3 on the *CDK14* promoter based on JASPAR and HOCOMOCO dataset (Fig. 6D). Further ChIP-qPCR showed that IRX3 was significantly enriched in the promoter region of *CDK14*, specifically between -808 and -796 (Fig. 6E). In addition to this, IRX3 depletion led to a notable decrease in the presence of IRX3 protein at the *CDK14* promoter (Fig. 6F). Then, we conducted a dual luciferase assay to ascertain whether IRX3 directly influences the transcriptional regulation of CDK14. Our findings indicated that the depletion of IRX3 significantly diminished the activity of the CDK14 promoter. However, it did not affect the activity of the promoter containing a HOX motif mutation (Fig. 6G). IRX3 binds to the genome and acts as a transcription factor, a role that is contingent upon its HOX domain. We constructed Flag-IRX3 full-length, Flag-IRX3 HOX deletion (IRX3-△HOX) plasmids and transfected them into 293FT cells. Overexpression of IRX3 significantly increased the expression of CDK14, but overexpression of IRX3-△HOX failed to induced this effect (Fig. 6H). All the results indicated that IRX3 binds to the promoter region of *CDK14*, thereby facilitating the activation of its transcription.

### IRX3 restrains the ubiquitination degradation of β-catenin through CDK14-mediated phosphorylation of LRP6

**Fig. 7:**
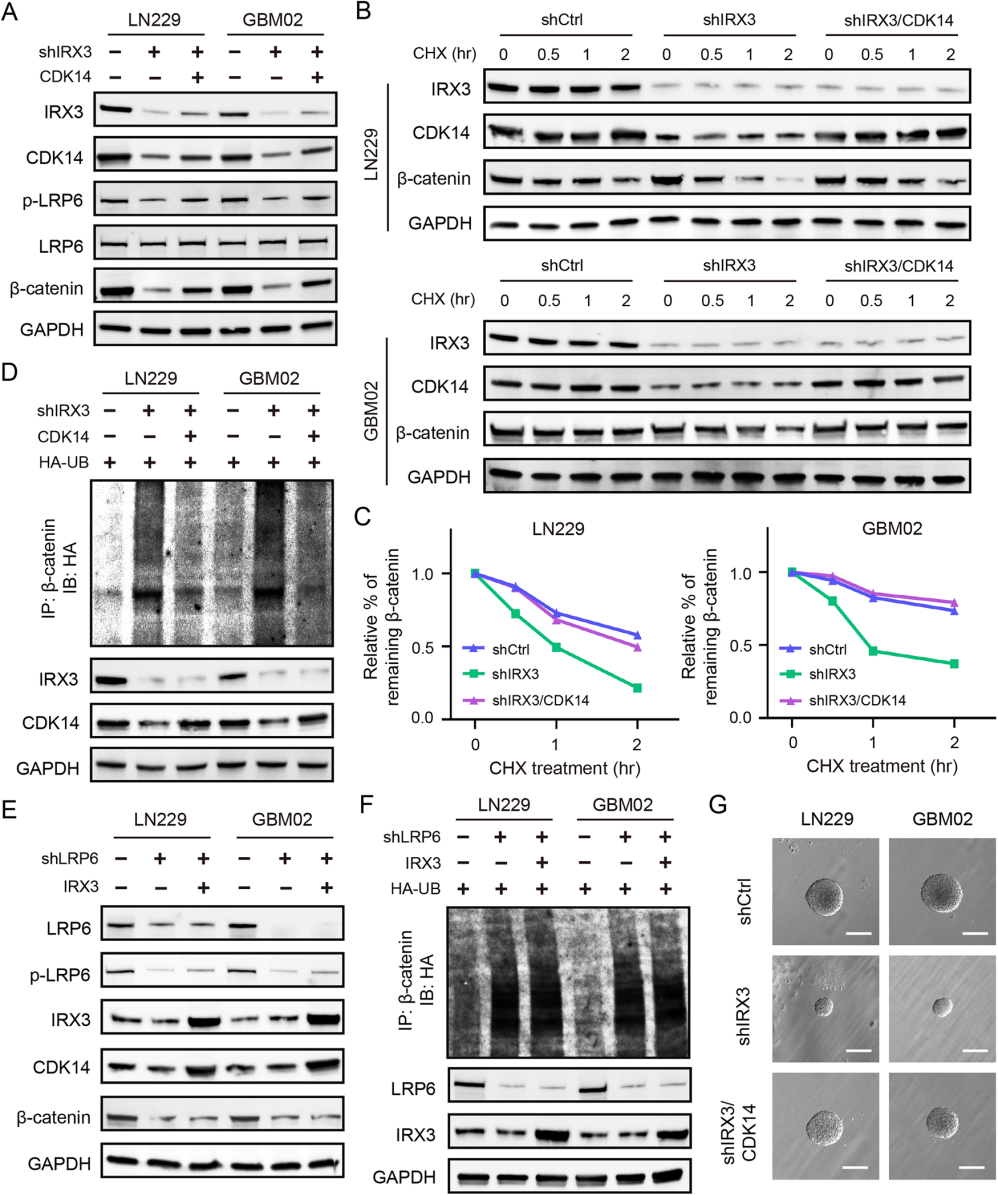
IRX3 restrains the ubiquitination degradation of β-catenin through CDK14-mediated phosphorylation of LRP6. **A** Immunoblotting analysis of the indicated proteins in IRX3-silenced GBM cells with or without reconstituted expression of CDK14. **B**, **C** Immunoblotting analysis of IRX3, CDK14 and β-catenin expressions in GBM cells expressing shCtrl, shIRX3, and shIRX3/CDK14 treated with or without CHX for the indicated times. The turnover rates of β-catenin were shown. **D** Ubiquitination assay of β-catenin in GBM cells expressing shCtrl, shIRX3, and shIRX3/CDK14 treated with MG132. Immunoblotting with the indicated antibodies was performed on equal amounts of cell lysates. **E** Immunoblotting analysis of the indicated proteins in LRP6-silenced GBM cells with or without IRX3 overexpression. **F** Ubiquitination assay of β-catenin in GBM cells expressing shCtrl, shLRP6, and shLRP6/IRX3 treated with MG132. Immunoblotting with the indicated antibodies was performed on equal amounts of cell lysates. **G** Representative microscopical images of colony formation in GBM cells expressing shCtrl, shLRP6, and shLRP6/IRX3.

To further verify whether IRX3 restrains the ubiquitination degradation of β-catenin by regulating CDK14, we restored CDK14 expression in IRX3-silenced cells. We found that the reconstituted expression of CDK14 rescued the phosphorylation level of LRP6 and expression of β-catenin (Fig. 7A). Turnover assay showed that the degradation rate of β-catenin induced by CHX treatment was significantly increased in IRX3-silenced GBM cells, and the increased degradation rate was mitigated by the reintroduction of CDK14 expression (Fig. 7B, C). The ubiquitination assay further verified that the enhancement of the ubiquitination-mediated degradation of β-catenin induced by IRX3 depletion was obviously reduced (Fig. 7D). In addition, we suppressed CDK14 activity in IRX3-overexpression GBM cells by using CDK14 inhibitor FMF-04-159-2. The results demonstrated that CDK14 inhibition abolished the IRX3 overexpression-induced increase in LRP6 phosphorylation and β-catenin expression (Supplementary Fig. S2A). In line with these results, the IRX3-mediated reduction of β-catenin ubiquitination and degradation was also reversed upon CDK14 inhibition (Supplementary Fig. S2B). The above results indicated that IRX3 restrains the ubiquitination degradation of β-catenin by regulating transcription of CDK14. CDK14 interacts with and phosphorylates LRP6, which is a critical co-receptor of Wnt, and subsequently inhibits phosphorylation and ubiquitination degradation of β-catenin. To determine whether LRP6 is essential for IRX3 to restrain the ubiquitination degradation of β-catenin, we constructed LRP6 knockdown GBM cells. Indeed, overexpression of IRX3 increased the expression of CDK14 but failed to stabilize β-catenin expression in LRP6-silenced GBM cells (Fig. 7E). Moreover, the ubiquitination assay demonstrated that overexpression of IRX3 could not rescue the ubiquitination-mediated degradation of β-catenin induced by LRP6 knockdown (Fig. 7F). All these results suggested that IRX3 restrains the ubiquitination degradation of β-catenin through CDK14-mediated phosphorylation of LRP6.

### IRX3-CDK14 axis promotes GBM growth by regulating Wnt/β-catenin pathway

**Fig. 8:**
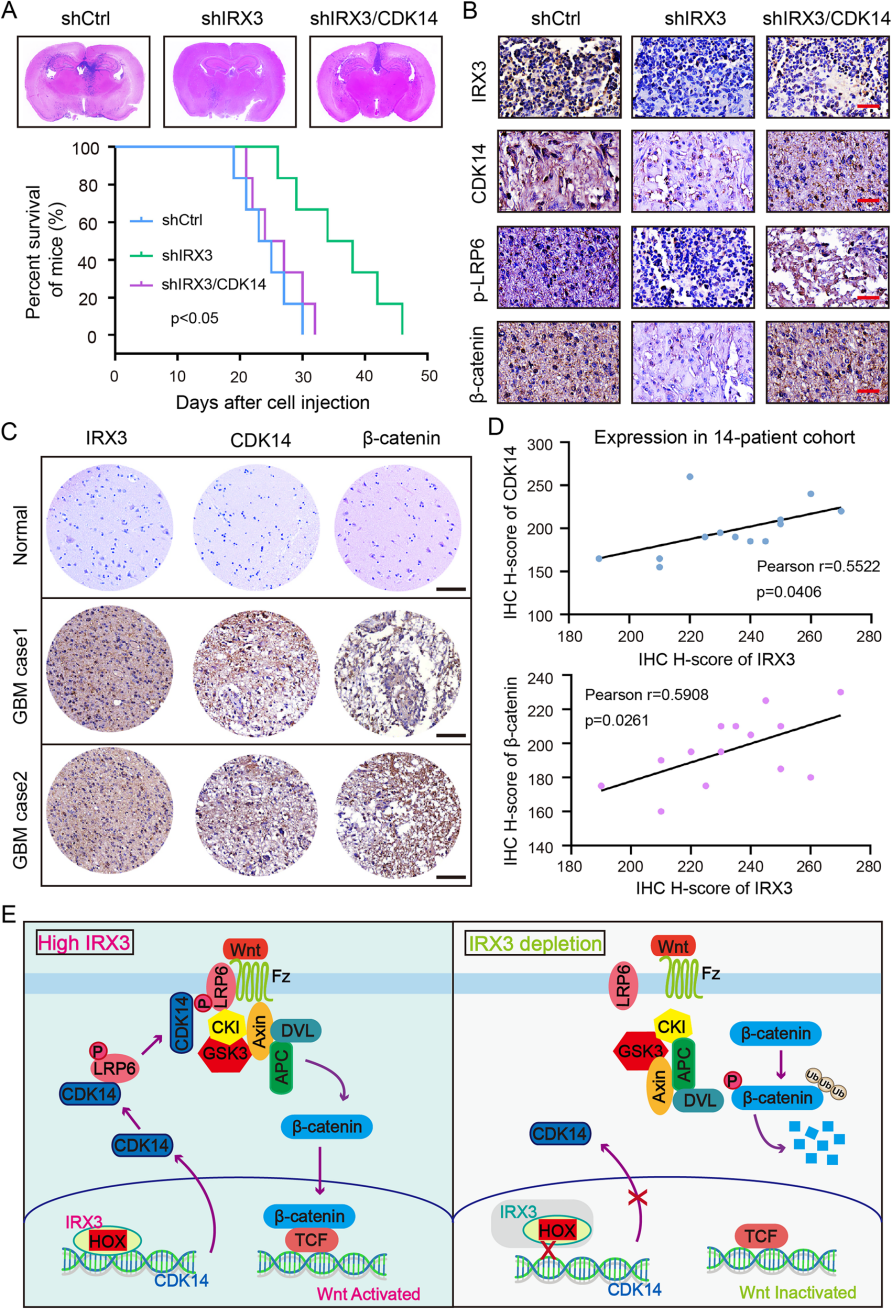
IRX3-CDK14 axis promotes GBM progression by regulating Wnt/β-catenin pathway. **A** Orthotopic tumorigenesis abilities of LN229 cells expressing shCtrl, shIRX3 or shIRX3/CDK14 and survival rates of mice (*n* = 6). **B** Immunohistochemical staining analysis of IRX3, CDK14, p-LRP6 and β-catenin in brain tumor tissues formed by LN229 cells expressing shCtrl, shIRX3 or shIRX3/CDK14. **C** Representative immunohistochemistry staining images of IRX3, CDK14 and β-catenin in normal brain tissues and GBM tissue samples. **D** Immunohistochemistry analyses of correlation between IRX3 and CDK14, β-catenin in 14 local patient cohort. IHC H-score was presented. **E** A schematic depicting the role of IRX3-CDK14-LRP6 axis in the regulation of Wnt/β-catenin pathway and GBM progression.

To examine the functional role of IRX3-CDK14 axis in the regulation of GBM growth, we firstly performed soft agar assay. The findings indicated that the inhibited ability of colony formation induced by IRX3 depletion was rescued by the reconstituted expression of CDK14, suggesting that IRX3 promotes the self-renewal ability of GBM cells by regulating CDK14 (Fig. 7G). Subsequently, we constructed animal models by injecting shCtrl, shIRX3, and shIRX3/CDK14 LN229 cells intracranially into nude mice. The results demonstrated that the depletion of IRX3significantly inhibited brain tumor growth and extended the survival duration in mice. Furthermore, these effects were reversed upon the re-establishment of CDK14 expression (Fig. 8A). IHC assay further showed that the reconstituted expression of CDK14 rescued the phosphorylation level of LRP6 and expression of β-catenin in tumor tissues of mice (Fig. 8B). In addition, we examined the expression of IRX3, CDK14 and β-catenin in GBM patients through performing IHC staining. The results indicated a significant positive correlation between the expression levels of IRX3 and both CDK14 and β-catenin in GBM tissue (Fig. 8C, D). Taken together, all of our findings indicated that IRX3 activates the transcription of CDK14 to phosphorylate LRP6 and restrain the ubiquitination degradation of β-catenin, thereby promoting GBM growth (Fig. 8E).

## Discussion

IRX genes belong to the TALE homeobox family and encode six related transcription factors (IRX1–IRX6), which control the development and cell differentiation of various human tissues [25, 26]. Recent studies have also identified and characterized the IRX family in the function and development of human cancers. IRX1 acts as a tumor suppressor that promotes differentiation into megakaryocytic and erythroid lineages, as well as induces growth arrest in ML-DS cells [27]. On the other hand, the up-regulation of IRX4 by histone 3 lysine 27 acetylation serves as a high risk of prostate cancer [28]. IRX5 promotes hepatocellular carcinoma progression by regulating de novo fatty acid synthesis reprogramming [29]. Therefore, the tumor-promoting or suppressing roles of IRX proteins are different in individual cancer types. Here, we demonstrated that IRX3 expression was elevated in GBM and was correlated with poor prognostic outcomes. Knockdown of IRX3 suppressed cell proliferation, migration, invasion, and tumor growth in GBM cells. Our finding identifies IRX3 as a potential prognostic biomarker and therapeutic target for the effective treatment of GBM.

GBM is recognized as the most aggressive and common form of primary brain cancer. Currently, the primary treatment modalities for patients diagnosed with GBM include surgical intervention, systemic chemotherapy and radiation therapy, as well as immunotherapy and targeted therapy [3, 5]. However, the 5-year survival rate for GBM patients remains suboptimal. GBM is distinguished by its complex molecular characteristics, which arise from alterations in various signaling pathways essential for cell proliferation, invasion, and resistance to therapeutic interventions, including EGFR/MEK/ERK, PI3K/AKT/mTOR, TGF-β, and NF-κB [30]. Besides these, the activation of Wnt signaling was also a common relevant and cryptic pathway involved in GBM growth and EMT. For example, Wnt10a, a member of Wnt family, remodels tumor microenvironment and promotes malignancy of glioblastoma by regulating JNK/c-Jun/FOSB pathway [31]. Inhibition of Wnt/β-catenin pathway by a novel Wnt inhibitor DK419 down-regulates MGMT expression and enhances GBM sensitivity to TMZ [32]. However, mechanisms of activation of Wnt/β-catenin signaling in GBM remain to be further investigated. In this study, we demonstrated that IRX3 stabilized the expression of β-catenin by restraining its ubiquitination degradation, thereby activated downstream genes including c-Myc, CCND1, WISP1, and c-Jun to promote GBM growth and EMT.

As a transcription factor, IRX3 does not directly stabilize β-catenin expression by regulating β-catenin deubiquitylation. Indeed, we identified that CDK14 was an important cooperator of IRX3 in regulating Wnt/β-catenin pathway and GBM progression. CDK14 is classified as a non-classical cyclin-dependent kinase (CDK) and belongs to the Cdc2 family, also referred to as PFTK1 (PFTAIRE protein kinase 1). This kinase is recognized for its regulatory functions in the cell cycle and its significant contributions to cell proliferation [33]. Recent studies have identified CDK14 as a promising tumor-associated gene, and further exploration of its biological function will provide new perspectives for studies on tumorigenesis and progression. In solid tumors, including breast cancer, non-small cell lung cancer, gastric cancer, and glioma, CDK14 functions as an oncogenic factor that promotes tumorigenesis and is correlated with unfavorable patient outcomes [34–37]. Our findings indicated that IRX3 facilitates the transcriptional activation of *CDK14* through direct interaction with the promoter region of the *CDK14* gene, specifically between -808 and -796. Reconstituted expression of CDK14 in IRX3-silenced GBM cells rescued the ubiquitination degradation of β-catenin induced by IRX3 knockdown. Our results indicated that IRX3 restrains the ubiquitination degradation of β-catenin by regulating transcription of CDK14 in GBM. Yao et al. reported that JNK1/2 phosphorylated IRX3 and led to its nuclear translocation for transcription [13]. In GBM, whether JNK can phosphorylate IRX3 to promote CDK14 transcription and stabilize β-catenin requires further investigation.

It has been shown that the CDK14/CCNY complex phosphorylates LRP6 at serine 1490, and phosphorylated LRP6 binds Frizzled and displaces GSK-3β from APC/Axin, which in turn stabilizes β-catenin expression and activates Wnt/β-catenin downstream gene transcription [38, 39]. LRP6 is a member of the cell surface and the low-density lipoprotein receptor superfamily [40] and serves as a crucial regulator of multiple physiological functions, including cell development, synaptic protection, cholesterol homeostasis, and the maintenance of energy balance [41, 42]. The intricate signaling pathway mediated by LRP6 is pivotal in modulating fundamental intracellular processes that drive tumor cell proliferation, migration, and stemness maintenance across a wide range of cancer types, including glioma [43, 44]. Our findings demonstrated that the silencing of IRX3 reduced the phosphorylation level of LRP6 in GBM cells. The effect was reversed by reconstituting the expression of CDK14. Furthermore, overexpression of IRX3 increased the expression of CDK14 but failed to restrain the ubiquitination degradation of β-catenin and promote the proliferation of LRP6-silenced GBM cells. Our findings demonstrated that IRX3 restrains the ubiquitination degradation of β-catenin through CDK14-mediated phosphorylation of LRP6.

In conclusion, our study demonstrates that IRX3 is significantly upregulated in GBM and drives GBM cell proliferation, migration, invasion and tumor growth. Mechanistically, IRX3 enhances CDK14 transcription by binding to its promoter, which in turn promotes LRP6 phosphorylation. This cascade suppresses ubiquitination-dependent β-catenin degradation, thereby activating the canonical Wnt/β-catenin signaling pathway and enhancing the expression of downstream target genes. Our data provide novel insights into the functional roles of IRX3-CDK14 axis in regulating Wnt/β-catenin signaling pathway and GBM progression, and identify that IRX3-CDK14 axis serves as a potentially effective target of GBM treatment.

## Supplementary information

Supplementary Figures

Reagents and antibodies

Primers

Original WB blots

## Data Availability

The data that support the findings of this study are available from the corresponding author upon reasonable request.
