# Supplementary material for: IRX3-CDK14 axis promotes glioblastoma progression by regulating LRP6-mediated canonical Wnt/β-catenin pathway
Source: Cell Death Dis. 2025 Dec 23;17(1):127. doi: 10.1038/s41419-025-08387-1 (PMC12847872; doi:10.1038/s41419-025-08387-1)
Supplement: Supplementary file 1 — Supplementary Figures [file 41419_2025_8387_MOESM1_ESM.docx]

**IRX3-CDK14 axis promotes glioblastoma progression by regulating LRP6-mediated canonical Wnt/β-catenin pathway**

**Supplementary Figures**

**Supplementary Figure S1**


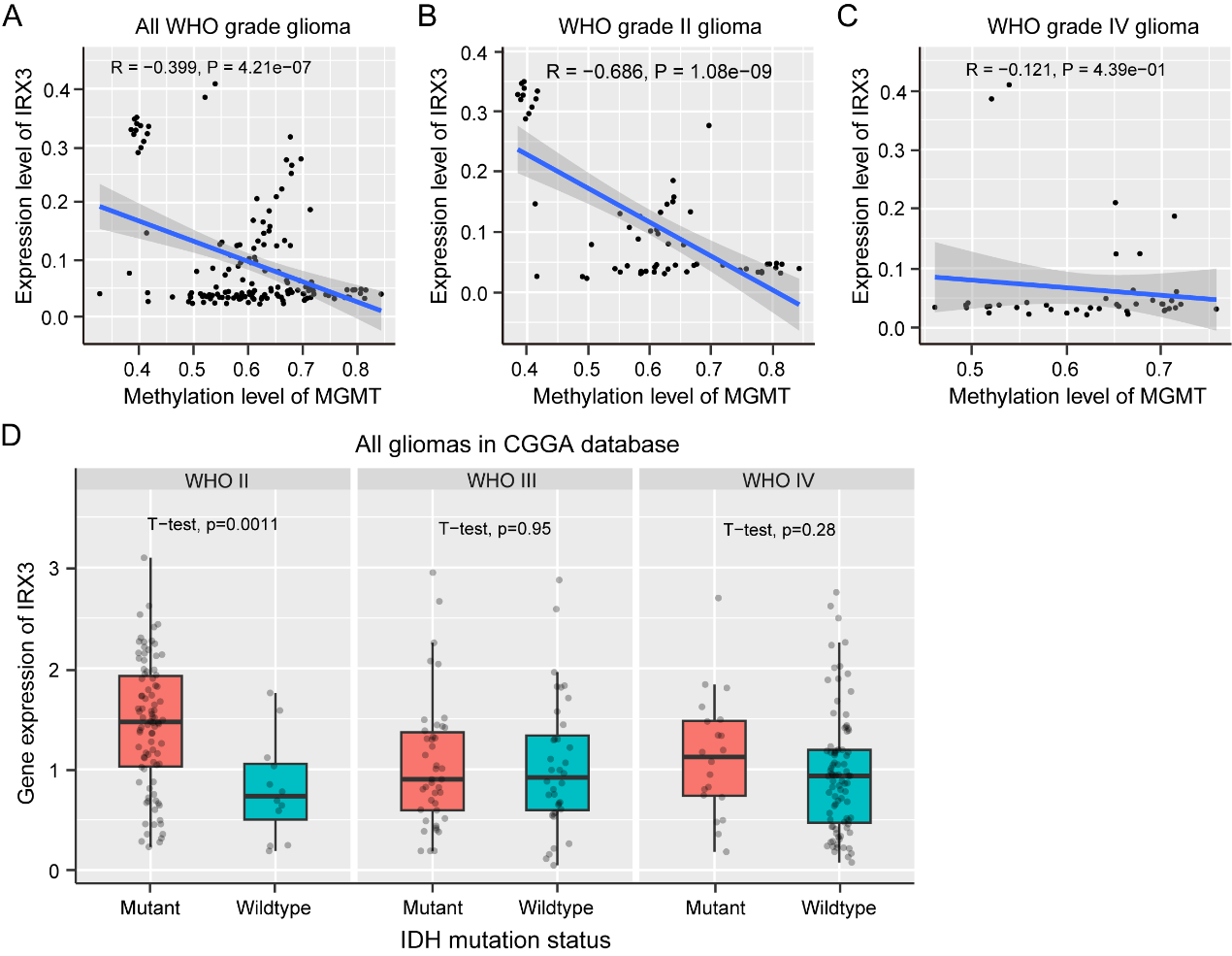


**Figure S1. Correlations between IRX3 expression and IDH mutation/MGMT promoter methylation status. (A-C)** Analysis of the correlation between IRX3 expression and MGMT promoter methylation status in low-grade gliomas (LGG) and high-grade gliomas (HGG) from the CGGA database. **(D)** Analysis of the correlation between IRX3 expression and IDH mutations in LGG and HGG from the CGGA database.

**Supplementary Figure S2**

**
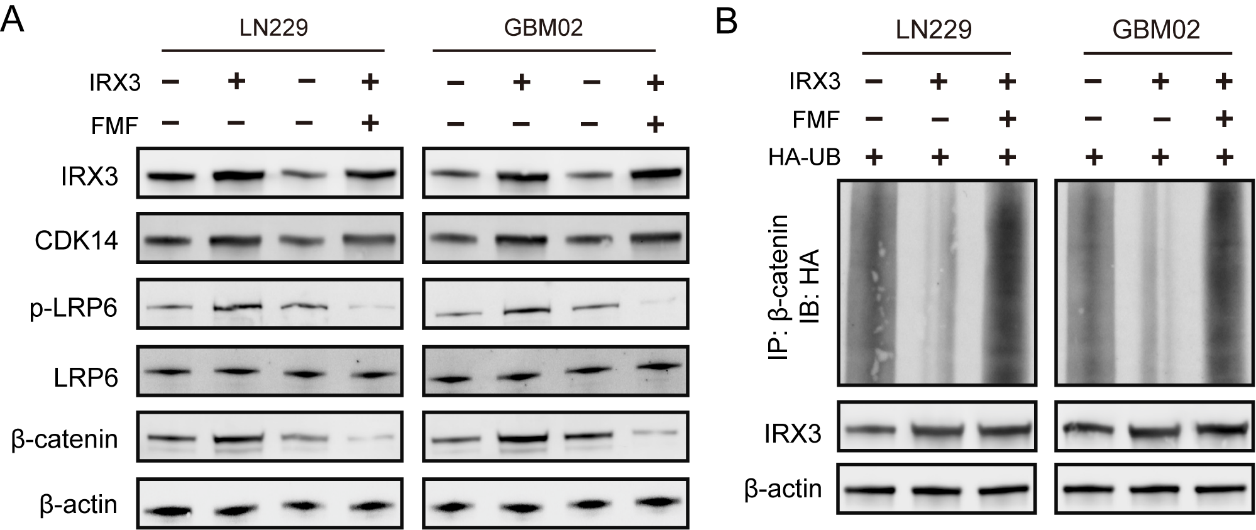
**

**Figure S2. Inhibition of CDK14 reversed IRX3-mediated reduction of β-catenin ubiquitination degradation. (A)** Immunoblotting analysis of the indicated proteins in IRX3-overexpressing GBM cells treated with or without CDK14 inhibitor FMF-04-159-2 (0.5μM). **(B)** Ubiquitination assay of β-catenin in GBM cells expressing empty vector or IRX3, which were pre-treated with or without FMF-04-159-2 prior to MG132 treatment. Immunoblotting with the indicated antibodies was performed on equal amounts of cell lysates.
