# Supplementary material for: IRX3-CDK14 axis promotes glioblastoma progression by regulating LRP6-mediated canonical Wnt/β-catenin pathway
Source: Cell Death Dis. 2025 Dec 23;17(1):127. doi: 10.1038/s41419-025-08387-1 (PMC12847872; doi:10.1038/s41419-025-08387-1)
Supplement: Supplementary file 2 — Reagents and antibodies [file 41419_2025_8387_MOESM2_ESM.docx]

**Table S1: Reagents and antibodies**

| **Reagents** | **Catalogue NO.** | **Company** |
| --- | --- | --- |
| Puromycin | 60209ES10 | YEASEN Biotechnology |
| Polybrene | sc-134220 | Santa Cruz Biotechnology |
| B27 | 17504044 | Gibco |
| G418 | 10131035 | Gibco |
| CCK-8 | C0041 | Beyotime |
| MG132 | HY-13259 | MedChemExpress |
| CHX | 239765 | Sigma-Aldrich |
| FMF-04-159-2 | HY-127104 | MedChemExpress |
| Clean-Blot IP | 21230 | Thermo Fisher |
| Protein A/G PLUS-Agarose | sc-2003 | Santa Cruz Biotechnology |
| **Antibodies** | **Catalogue NO.** | **Company** |
| IRX3 | sc-166657X | Santa Cruz |
| GAPDH | 60004-1-Ig | Proteintech |
| β-catenin | 51067-2-AP | Proteintech |
| N-cadherin | 22018-1-AP | Proteintech |
| E-cadherin | 20874-1-AP | Proteintech |
| Fibronectin | 15613-1-AP | Proteintech |
| MMP9 | 10375-2-AP | Proteintech |
| Vimentin | 10366-1-AP | Proteintech |
| CDK14 | 21612-1-AP | Proteintech |
| HA-Tag | 3724 | CST |
| LRP6 | 3395 | CST |
| p-LRP6 | 2568 | CST |
| Flag-Tag | F9291 | Sigma-Aldrich |
