# Supplementary material for: IRX3-CDK14 axis promotes glioblastoma progression by regulating LRP6-mediated canonical Wnt/β-catenin pathway
Source: Cell Death Dis. 2025 Dec 23;17(1):127. doi: 10.1038/s41419-025-08387-1 (PMC12847872; doi:10.1038/s41419-025-08387-1)
Supplement: Supplementary file 3 — Primers [file 41419_2025_8387_MOESM3_ESM.docx]

**Table S2** Primers and shRNA sequences used in this study

Primers for qRT-PCR

| Primer name 5′-3 | 5′-3′ |
| --- | --- |
| IRX3-F  IRX3-R  GAPDH-F | GAGGGAAACGCTTATGGGAGC  CGCCGTCTAAGTTCTCCAAATC  AACGGATTTGGTCGTATTGGG |
| GAPDH-R | CCTGGAAGATGGTGATGGGAT |
| c-Myc-F | ACACTAACATCCCACGCTCTG |
| c-Myc-R | CGCATCCTTGTCCTGTGAGT |
| c-Jun-F  c-Jun-R | GGAGGGAGGTTTGTGAGAGC  ACAAACAACACTGGGCAGGA |
| CCND1-F  CCND1-R  WISP1-F  WISP1-R  CDK14-F  CDK14-R  β-catenin-F  β-catenin -R | GCTGCGAAGTGGAAACCATC  CCTCCTTCTGCACACATTTGAA  AGGAACTGCATAGCCTACACA  TGGTACACAGCCAGACACTTC  TGGACCAGTTTGGGGAAGTTG  ATTCCCTGGCAGTTCCGTG  CTGAGGAGCAGCTTCAGTCC  CCATCAAATCAGCTTGAGTAGCC |

Primers for ChIP assay

| Primer name 5′-3 | 5′-3′ |
| --- | --- |
| CDK14-F (-1472/-1349) | TGCTCCCCACTTAGCTTGAC |
| CDK14-R (-1472/-1349)  CDK14-F (-928/-788) | GCCCCCTGCCTTCCTTAAAT  ACCAAGGGCTGTGTCTGCTAT |
| CDK14-R (-928/-788) | GCGGAAGCTTCAACTGTAACC |
| CDK14-F (-721/-561) | CCACCTTTCCCCTTGGATCT |
| CDK14-R (-721/-561) | TCCACGAGCGTGTGTGAAAA |

Sequences of shRNA

| Target Sequence | 5′-3′ |
| --- | --- |
| shIRX3#1-F | CCGGTTGTAAGCATGTCCGTGTATACTCGAGTATACACGGACATGCTTACAATTTTTG |
| shIRX3#1-R | AATTCAAAAATTGTAAGCATGTCCGTGTATACTCGAGTATACACGGACATGCTTACAA |
| shIRX3#2-F | CCGGGCGCCTCAAGAAGGAGAATAACTCGAGTTATTCTCCTTCTTGAGGCGCTTTTTG |
| shIRX3#2-R | CCGGGCGCCTCAAGAAGGAGAATAACTCGAGTTATTCTCCTTCTTGAGGCGCTTTTTG |
| shLRP6-F | CCGGCCGAATTTATTGGACTGATATCTCGAGATATCAGTCCAATAAATTCGGTTTTTG |
| shLRP6-R | AATTCAAAAACCGAATTTATTGGACTGATATCTCGAGATATCAGTCCAATAAATTCGG |
