# Supplementary material for: IRX3-CDK14 axis promotes glioblastoma progression by regulating LRP6-mediated canonical Wnt/β-catenin pathway
Source: Cell Death Dis. 2025 Dec 23;17(1):127. doi: 10.1038/s41419-025-08387-1 (PMC12847872; doi:10.1038/s41419-025-08387-1)

Fig.1E-IRX3

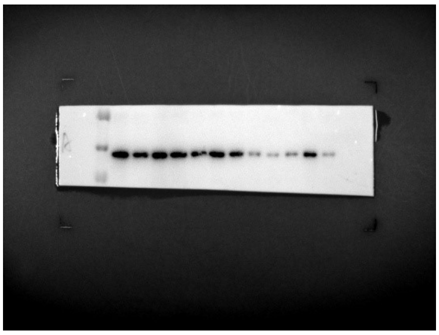

Fig.1E-GAPDH

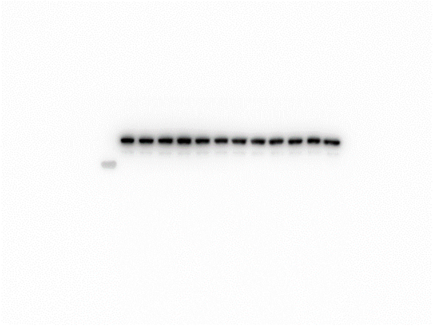

Fig.1F-IRX3

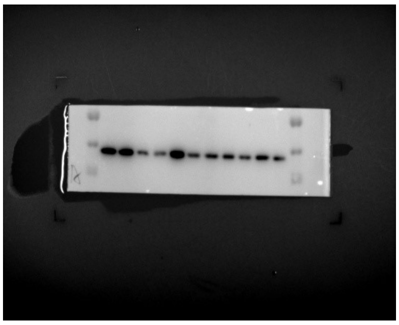

Fig.1F-GAPDH

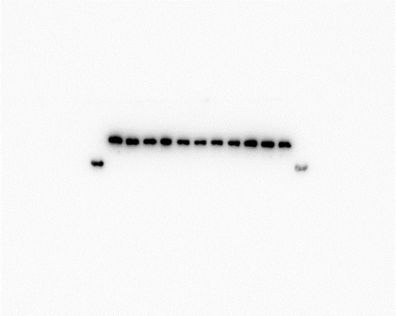

Fig.2A and B -IRX3

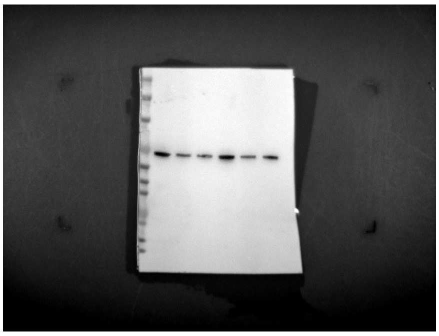

Fig.2A and B -GAPDH

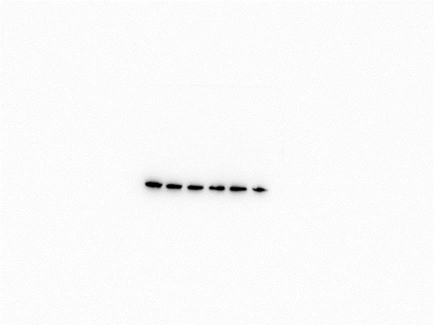

Fig.3C-IRX3

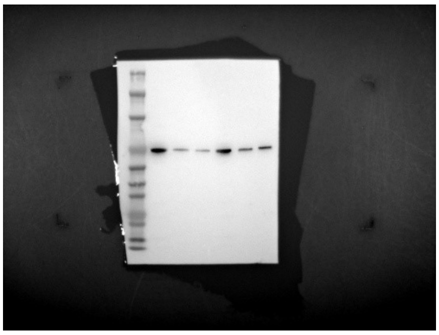

Fig.3C-β-catenin

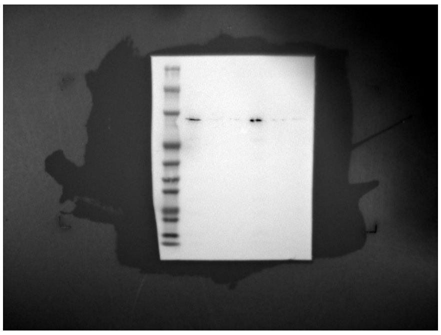

Fig.3C-GAPDH

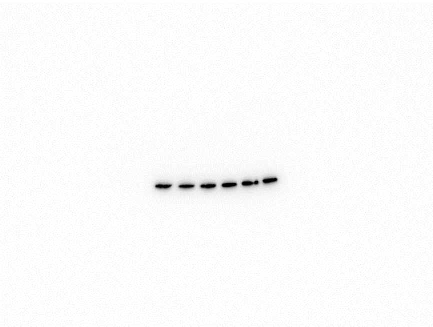

Fig.3F-N-cadherin

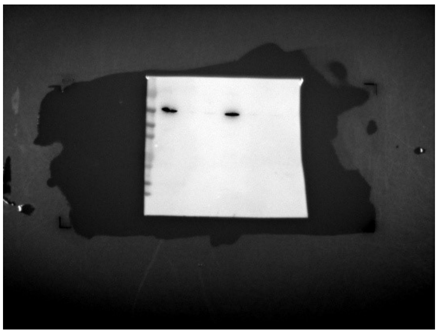

Fig.3F-E-cadherin

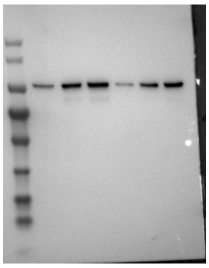

Fig.3F-Fibronectin

Fig.3F-Vimentin

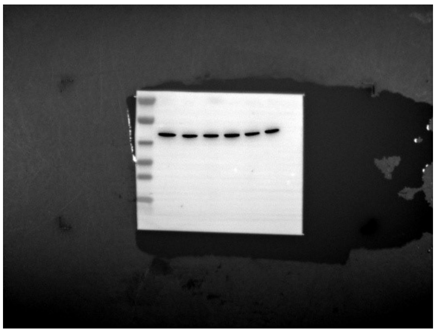

Fig.3F-MMP9

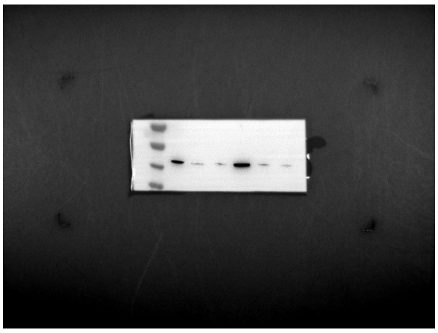

Fig.3F-GAPDH

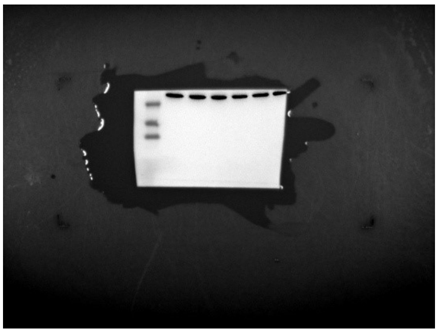

Fig.4A-IRX3

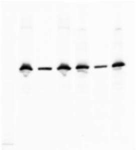

Fig.4A-GAPDH

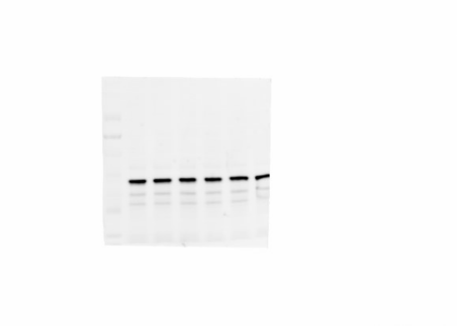

Fig.4F-β-catenin

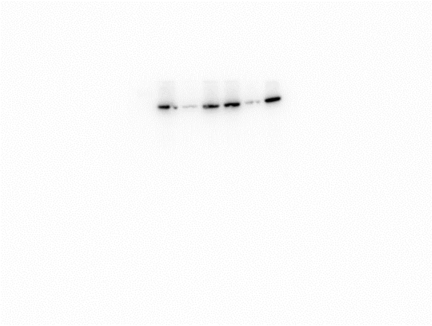

Fig.4F-N-cadherin

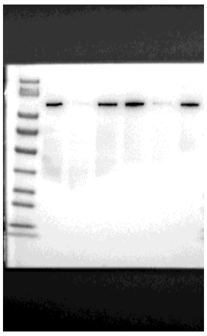

Fig.4F-E-cadherin

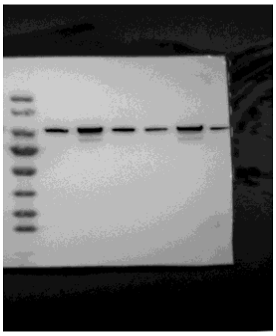

Fig.4F-Fibronectin

Fig.4F-MMP9

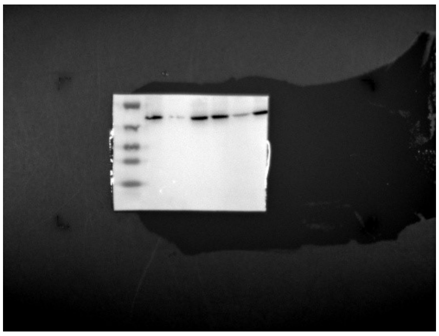

Fig.4F-GAPDH

Fig.5B-IRX3

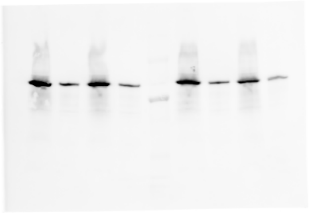

Fig.5B-GAPDH

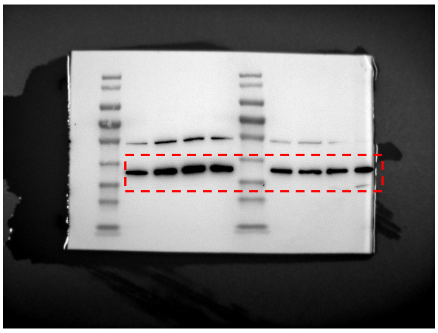

Fig.5B-β-catenin

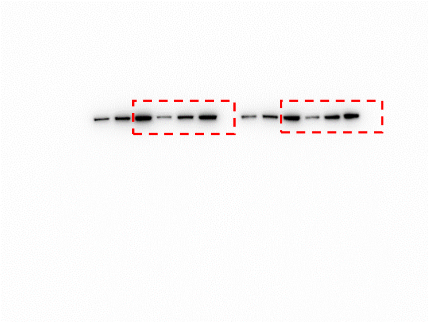

Fig.5B-GAPDH (2)

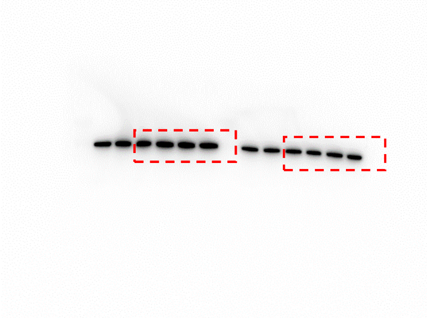

Fig.5C-LN229-IRX3

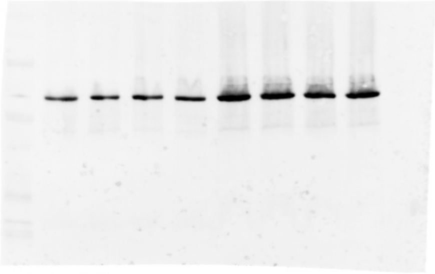

Fig.5C-LN229-β-catenin

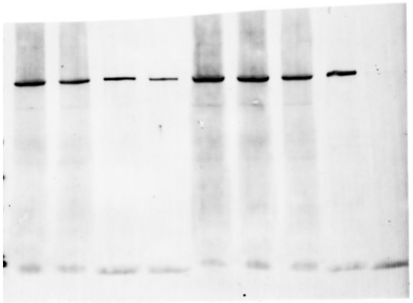

Fig.5C-LN229-GAPDH

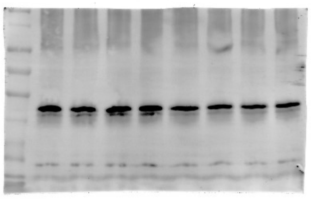

Fig.5C-GBM02-IRX3

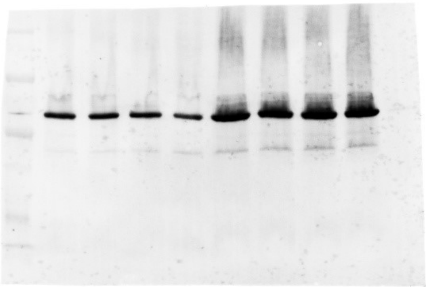

Fig.5C-GBM02-β-catenin

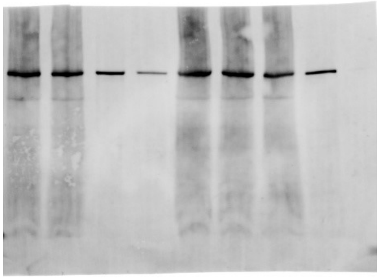

Fig.5C-GBM02-GAPDH

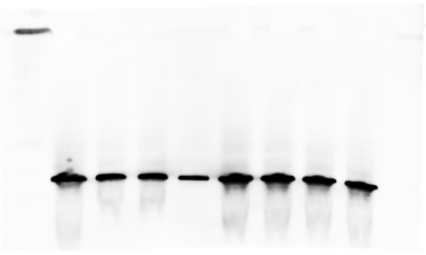

Fig.5E-β-catenin

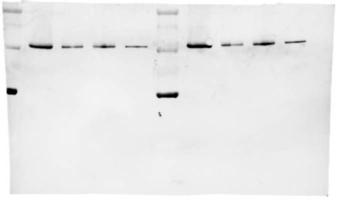

Fig.5E-GAPDH

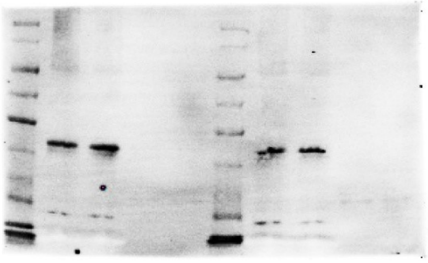

Fig.5E-Lamin A

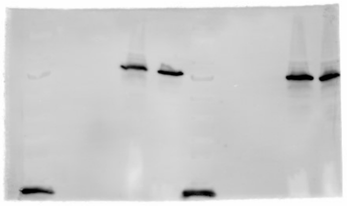

Fig.5F-IRX3

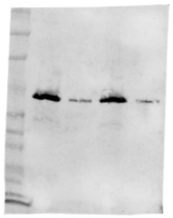

Fig.5F-GAPDH

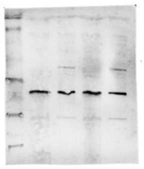

Fig.5F-HA

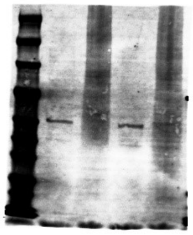

Fig.6C-IRX3

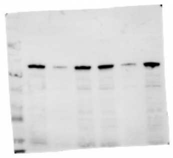

Fig.6C-CDK14

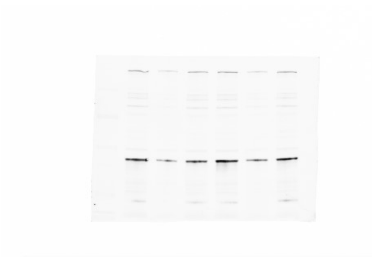

Fig.6C-GAPDH

Fig.6C-p-LRP6

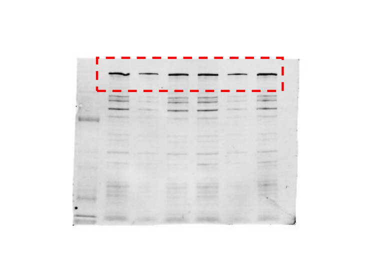

Fig.6C-LRP6

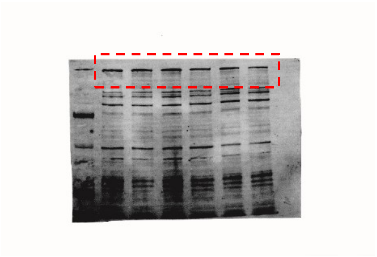

Fig.6H-Flag

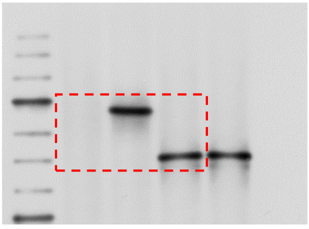

Fig.6H-CDK14

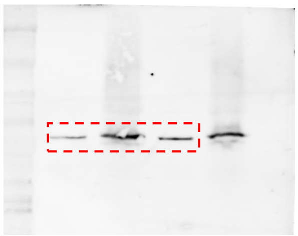

Fig.6H-GAPDH

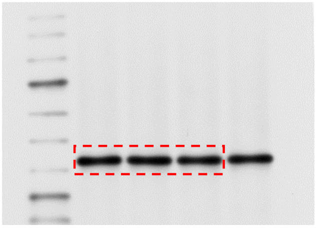

Fig.7A-IRX3

Fig.7A-CDK14

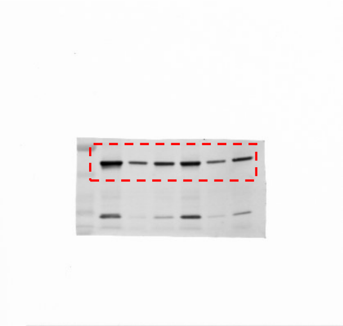

Fig.7A-p-LRP6

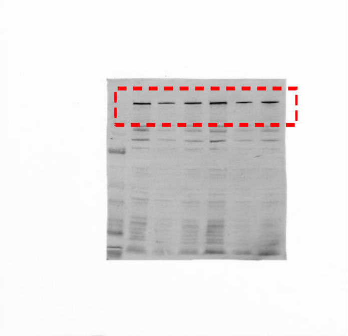

Fig.7A-LRP6

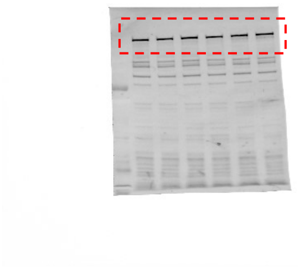

Fig.7A-β-catenin

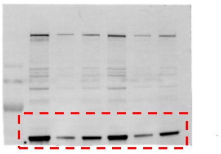

Fig.7A-GAPDH

Fig.7B-LN229-IRX3

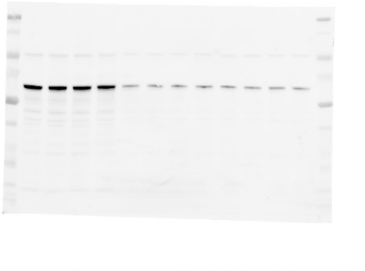

Fig.7B-LN229-CDK14

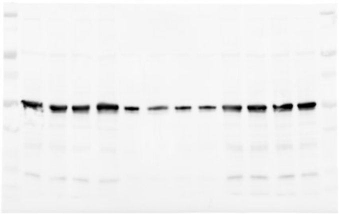

Fig.7B-LN229-β-catenin

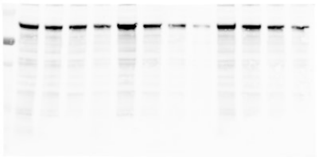

Fig.7B-LN229-GAPDH

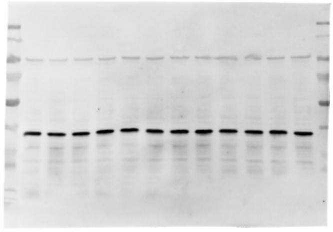

Fig.7B-GBM02-IRX3

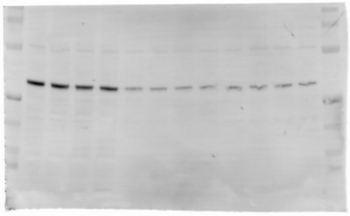

Fig.7B-GBM02-CDK14

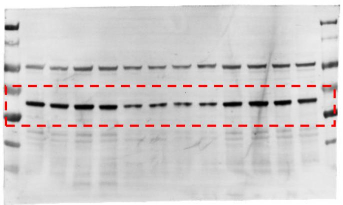

Fig.7B-GBM02-β-catenin

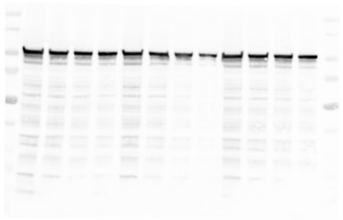

Fig.7B-GBM02-GAPDH

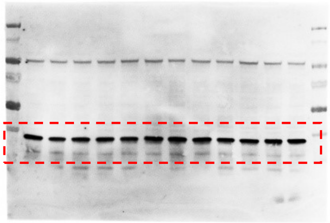

Fig.7D-IRX3

Fig.7D-CDK14

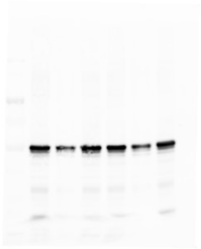

Fig.7D-HA

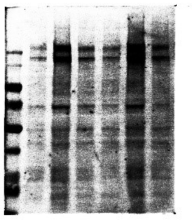

Fig.7D-GAPDH

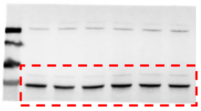

Fig.7E-LRP6

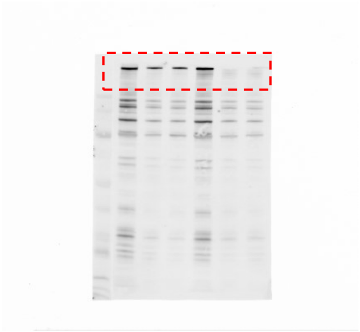

Fig.7E-p-LRP6

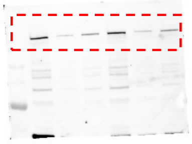

Fig.7E-IRX3

Fig.7E-CDK14

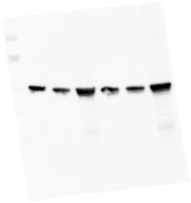

Fig.7E-β-catenin

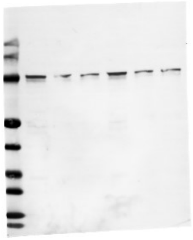

Fig.7E-GAPDH

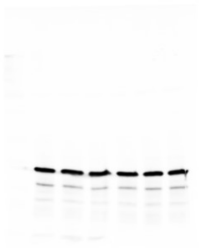

Fig.7F-LRP6

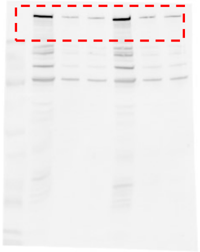

Fig.7F-IRX3

Fig.7F-HA

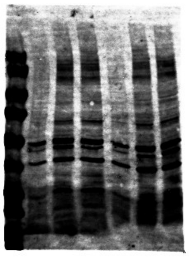

Fig.7F-GAPDH

Fig.S2A-IRX3

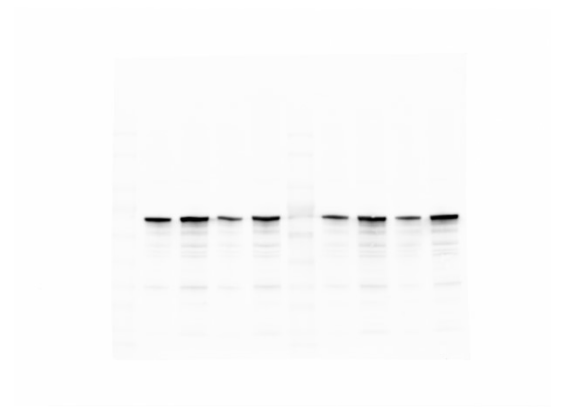

Fig.S2A-CDK14

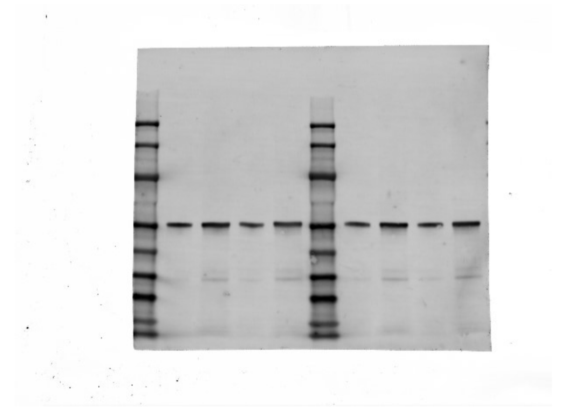

Fig.S2A-p-LRP6

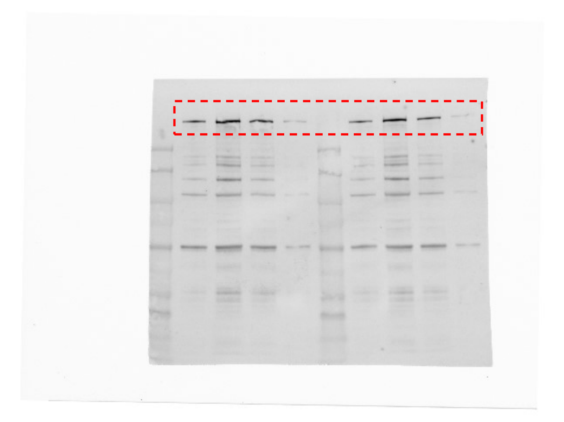

Fig.S2A-LRP6

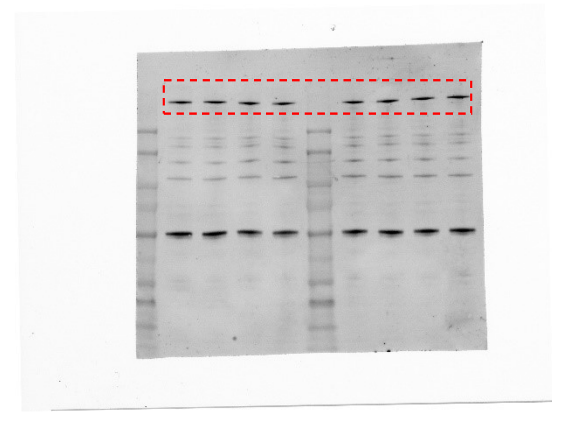

Fig.S2A-β-catenin

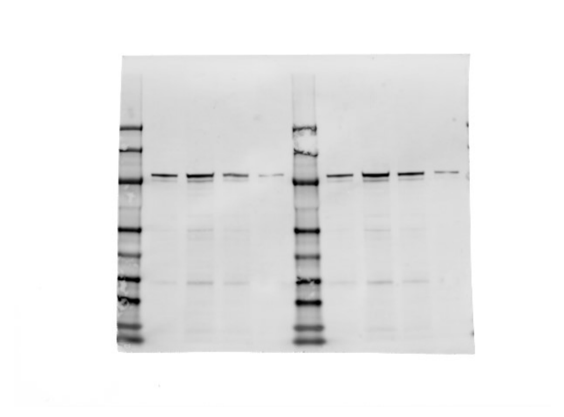

Fig.S2B-β-actin

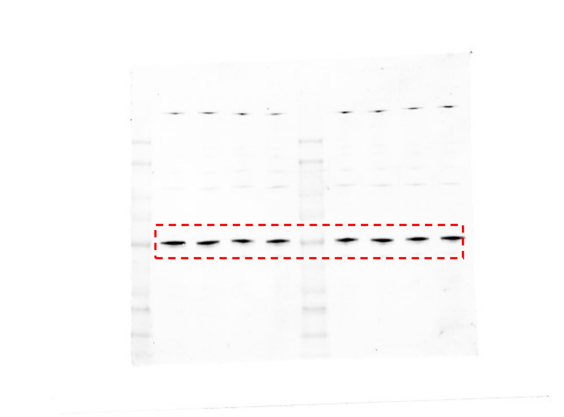

Fig.S2B-IRX3

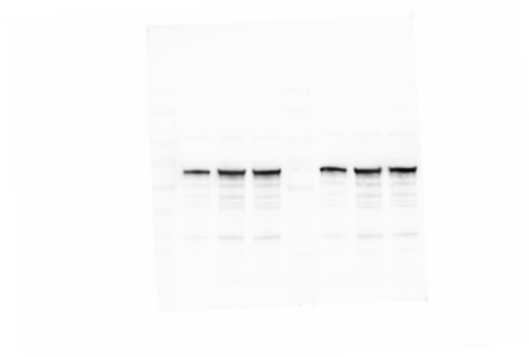

Fig.S2B-HA

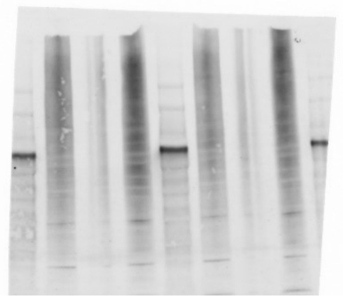

Fig.S2B-β-actin

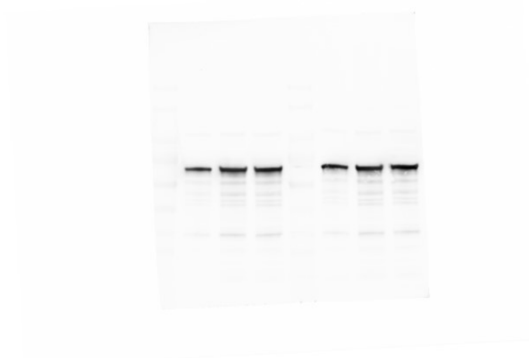

Supplement: Supplementary file 4 — Original WB blots [file 41419_2025_8387_MOESM4_ESM.pdf]
